# Oncolytic Parapoxvirus induces Gasdermin E-mediated pyroptosis and activates antitumor immunity

**DOI:** 10.1038/s41467-023-35917-2

**Published:** 2023-01-14

**Authors:** Jing Lin, Shihui Sun, Kui Zhao, Fei Gao, Renling Wang, Qi Li, Yanlong Zhou, Jing Zhang, Yue Li, Xinyue Wang, Le Du, Shuai Wang, Zi Li, Huijun Lu, Yungang Lan, Deguang Song, Wei Guo, Yujia Chen, Feng Gao, Yicheng Zhao, Rongrong Fan, Jiyu Guan, Wenqi He

**Affiliations:** 1 Key Laboratory of Zoonosis Research, Ministry of Education, College of Veterinary Medicine, Jilin University, 130062 Changchun, China; 2 Department of Laboratory Animals, College of Animal Science, Jilin University, 130062 Changchun, China; 3 Department of Hematology, The first hospital of Jilin University, 130021 Changchun, China; 4 Department of Gastrointestinal Surgery, The first hospital of Jilin University, 130021 Changchun, China; 5 Changchun University of Chinese Medicine, 130017 Changchun, China; 6 Department of Biosciences and Nutrition, Karolinska Institutet, 14183 Huddinge, Sweden

**Keywords:** Tumour vaccines, Viral host response, Cancer microenvironment, Tumour immunology

## Abstract

The advantage of oncolytic viruses (OV) in cancer therapy is their dual effect of directly killing tumours while prompting anti-tumour immune response. Oncolytic parapoxvirus ovis (ORFV) and other OVs are thought to induce apoptosis, but apoptosis, being the immunogenically inert compared to other types of cell death, does not explain the highly inflamed microenvironment in OV-challenged tumors. Here we show that ORFV and its recombinant therapeutic derivatives are able to trigger tumor cell pyroptosis via Gasdermin E (GSDME). This effect is especially prominent in GSDME-low tumor cells, in which ORFV-challenge pre-stabilizes GSDME by decreasing its ubiquitination and subsequently initiates pyroptosis. Consistently, GSDME depletion reduces the proportion of intratumoral cytotoxic T lymphocytes, pyroptotic cell death and the success of tumor ORFV virotherapy. In vivo, the OV preferentially accumulates in the tumour upon systemic delivery and elicits pyroptotic tumor killing. Consequentially, ORFV sensitizes immunologically ‘cold’ tumors to checkpoint blockade. This study thus highlights the critical role of GSDME-mediated pyroptosis in oncolytic ORFV-based antitumor immunity and identifies combinatorial cancer therapy strategies.

## Abstract (web summary)

Oncolytic viruses are able to target tumours and thought to induce apoptosis while remodelling the tumour immune microenvironment. Here authors show in an oncolytic parapoxvirus ovis model that pyroptosis, a highly immunogenic Gasdermin-E-dependent cell death mechanism, is the dominant cell death pathway during virotherapy.

## Introduction

Oncolytic viruses (OV) are compelling agents for cancer therapy, they are native or engineered viruses that selectively target and kill tumors^1,2^. OV-executed tumor inhibition is mainly through direct oncolytic effect and immune cell-mediated tumor clearance^2^. As a kind of novel immunotherapy, OV-based therapy is considered to induce significant antitumor immunity, either through innate or adaptive immune cells^3^. Notably, a promising approach for harnessing OVs is to improve the efficacy of immune checkpoint inhibition (ICI), which is dependent on the infiltration and activation of cytotoxic T lymphocytes (CTL) within tumor lesions^2,4^. Given that a majority of cancer patients were initially refractory to ICI therapy^5,6^, the approach of combining with OVs is of great clinical significance. It is believed that tumors with high immunogenic properties recruit more CTLs into the tumor microenvironment and display more effective responses to ICIs^7^. Armed OVs expressing pro-inflammatory cytokines as well as even unmodified OVs can release tumor-associated antigens (TAA) and damage-associated molecular patterns (DAMP) from infected tumors, make the tumors to display immunogenicity and thereby induce antigen-specific CD8^**+**^ T cells^1,2,8^. Despite of the encouraging potential of OVs and that various OVs already in clinical studies, the clinical outcomes of OVs monotherapies and combinational treatments with ICIs still need to be improved^5^. Therefore, uncovering the immune-stimulating mechanisms of OVs and further optimizing therapeutic strategies are becoming priority issues.

Oncolytic Parapoxvirus ovis (ORFV) is a promising OV with high immunogenicity and unique immune-stimulating properties. As an antitumor biotherapeutic candidate, ORFV has limited pathogenicity in humans^9^. Thus, generating attenuated ORFV, such as one or more virulence gene deletion recombinants, may have better clinically acceptable safety. Additionally, although the production of neutralizing antibodies was challenging the application of other OVs, but had no effect on the repeated use of ORFV^9^. Despite the above merits, whether and how ORFV can induce immunogenic cell death (ICD) are not fully elucidated. Traditionally, ORFV and other OVs are thought to induce cell apoptosis, as caspase 3 is activated in the context of OV challenge^10–13^. However, apoptosis is believed to be immunologically quiescent^14,15^, its noninflammatory characteristic contradicts with the pro-inflammatory and immunogenic properties of OVs. Of note, a pivotal finding indicated that caspase 3 activation can also cleave Gasdermin E (GSDME) and release the pore-forming GSDME-N fragment to induce pro-inflammatory cell death-pyroptosis^16^.

Pyroptosis is an inflammatory cell death that features cell swelling, membrane rupture, and cellular immunostimulatory content release^16–18^. Pyroptosis is regarded as gasdermin-mediated programmed cell death^18^. Six members of gasdermins have been reported in humans, and most gasdermins except for DFNB59 have flexible linkers connected N-terminal pore-forming domain to the C-terminal inhibitory domain^10^. Gasdermin D (GSDMD) is the most studied gasdermin, which is the substrate of pro-inflammatory caspases (caspase-1, -4, -5, and -11). Caspase-mediated cleavage of the linker liberates the pore-forming domain to disrupt the cell membrane and trigger pyroptosis^16–18^. GSDME is another well-characterized gasdermin, it is recognized and cleaved by active caspase 3 and killer cell-released granzyme B (GZMB) under certain stress^16,19^. Notably, GSDME-mediated pyroptosis play a critical role in the context of antitumor immunity. Spontaneous pyroptosis in GSDME-expressing tumors has been shown to activate the tumor immune responses and cause ICD^19^. However, the expression level of GSDME is regarded as a determinant in caspase 3-dependent cell pyroptosis, as cells with low GSDME levels switch to apoptosis instead of pyroptosis upon stimulations^16^. Coincidentally, *GSDME* is often suppressed in many kinds of cancers^16,20–22^, which impedes the efficacy of anticancer therapies such as chemo- or immunotherapy^23,24^. Meanwhile, exogenous GSDME overexpression or decitabine-induced GSDME improved antitumor effects^16,19^. Therefore, identifying strategies for elevating GSDME levels in tumors to further accelerate GSDME-mediated cell pyroptosis are promising approaches for effective tumor clearance.

Here we explore whether oncolytic ORFV can induce tumor cell pyroptosis and the underlying mechanism of ORFV-induced pyroptosis. We observe that ORFV treatment can induce GSDME-mediated tumor cell pyroptosis both in vitro and in vivo. Importantly, we further study the mechanisms of ORFV-triggered pyroptosis in GSDME-low cells. Next, we explore the importance of GSDME in the process of ORFV-induced tumor shrinkage and CTL recruitment. By harnessing the oncolytic ORFV-based strategy, we generate different ORFV recombinants to study tumor-targeting and killing effects in mouse models. Moreover, we combine checkpoint blockade or chemotherapy with oncolytic ORFV to achieve synergistic antitumor effects, which underscore the translational relevance of our findings.

## Results

### Oncolytic ORFV triggers GSDME-mediated pyroptosis in GSDME-expressing tumor cells

**Fig. 1:**
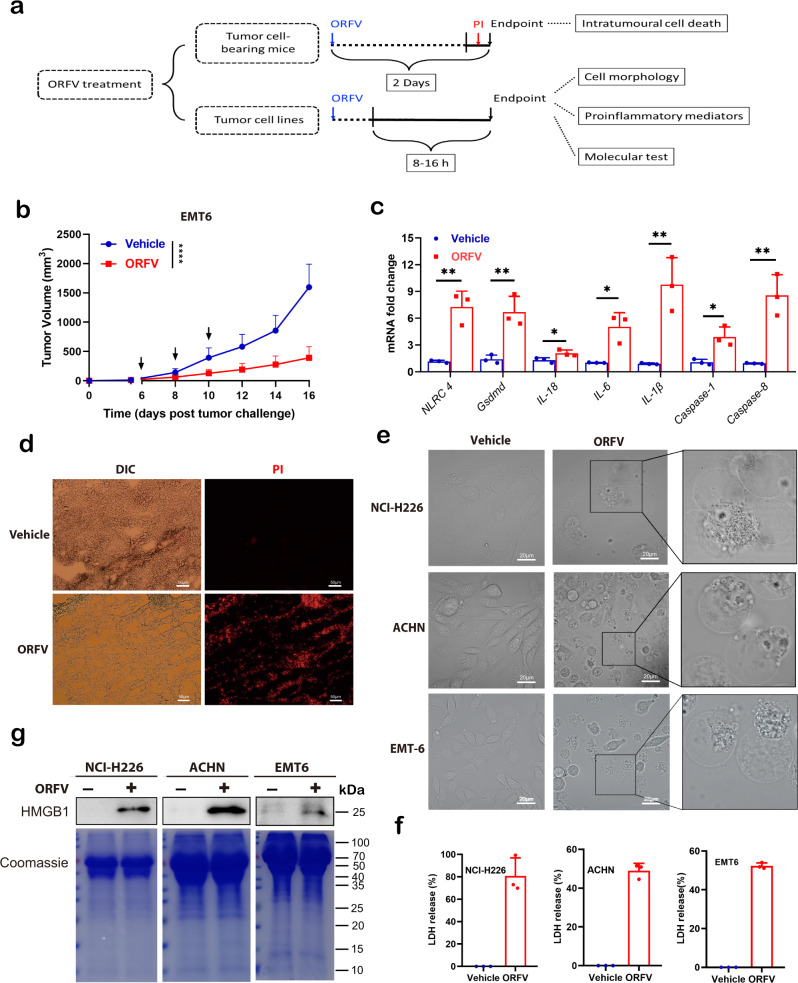
ORFV triggers tumor pyroptosis. **a** Schematic illustration of experimental design. Mice were engrafted with murine tumor cells, followed by intratumorally (i.t.) ORFV challenge. Tumor volume, intratumoral lytic cell death, and pro-inflammatory signaling were determined. Human tumor tissues and tumor cell lines were treated with ORFV for indicated time points, then cell morphology, pro-inflammatory mediators, and molecular test were performed. **b** Six weeks old female BALB/c mice were s.c. engrafted with EMT6 cells (1 × 10^6^ cells/mouse) and i.t. treated with ORFV (1 × 10^5^ TCID_50_/mouse) (*n* = 8–10) (*P* < 0.0001). **c** RT-qPCR analysis from ORFV or vehicle-treated EMT6 tumor tissues (*n* = 3). Relative mRNA level changes of selected inflammatory genes were displayed (*NLRC4*: *P* = 0.0041, *Gsdmd*: *P* = 0.0130, *IL-18*: *P* = 0.0416, *IL-6*: *P* = 0.0123, *IL-1β*: *P* = 0.0071*, Caspase-1*: *P* = 0.0005, *Caspase-8*: *P* = 0.0048). **d** Images acquired with a fluorescence microscope display PI uptake in in situ EMT6 tumor tissues after the challenge of ORFV (1 × 10^5^ TCID_50_/mouse) for 2 days (scale bar: 50 μm). Mice were intravenously (i.v.) administrated with PI dye 2 h before the euthanasia of animals. DIC differential interference contrast, a kind of bright field. **e** Images acquired by microscopy display changes in NCI-H226, ACHN, and EMT6 cell morphology after ORFV treatment (MOI = 1) for 16 h (scale bar: 20 μm). **f** LDH release assays were performed with NCI-H226, ACHN and EMT6 cells after ORFV (MOI = 1) treatment (*n* = 3). **g** HMGB1 level detection in supernatants from ORFV or vehicle-treated NCI-H226, ACHN, and EMT6 cells. Coomassie staining is shown as the control. The above experiments were successfully repeated two to three times. *P < 0.05, **P < 0.01, and ****P < 0.0001. Two-tailed unpaired Student’s *t*-tests were performed for the statistical analyses in (**b**, **c**), and the results are presented as the mean ± SD.

Given that several OVs are highly immunogenic, with the ability of recruiting and activating immune cells, and especially that oncolytic ORFV can recruit cytotoxic natural killer (NK) cells to the target tumor niche^1,9,25^, we explored whether and how ORFV was able to trigger ICD and further induce pro-inflammatory status in in situ tumors, ex vivo tumors and in vitro tumor cells. ORFV treatment was performed as shown in the schematic diagram (Fig. 1a). ORFV is highly epitheliotropic, which can propagate in primary ovine fetal turbinate (OFTu) cells (Supplementary Fig. 1). Thus, epithelium-derived murine breast cancer cell EMT6 was engrafted into the mice for examining the pro-inflammatory effect of ORFV. We observed that ORFV intratumoral treatment (10^5^ TCID_50_) not only suppressed tumor growth as expected, but also induced the mRNA expression of several pro-inflammatory genes, as compared to vehicle (PBS) treatment (Fig. 1b, c, Supplementary Fig. 2a, b, and Supplementary Table 1). Of note, the analysis revealed that pyroptosis-related genes (*Gsdmd, Casp1, Casp8, Il1b,* and *Il18*) were involved in reshaping the intratumoral pro-inflammatory environment. Additionally, when EMT6 tumor-bearing mice were administered with propidium iodide (PI), which could indicate pyroptotic cell death in vivo^26^, we found that ORFV treatment can induce lytic cell death (PI-positive signal) in part of tumor cells (Fig. 1d). The above data suggest that ORFV induced inflammatory tumor cell death.

To determine whether ORFV-triggered cell death is pyroptotic, epithelium-derived tumor cell lines like human cancer cell NCI-H226 and ACHN, as well as murine cancer cell EMT6 and CT26, were treated with ORFV (multiplicity of infection (MOI) = 1) in vitro (Fig. 1a). We have observed that ORFV-treated tumor cells underwent cell swelling with bubbles blowing from cell membranes (Fig. 1e). By detecting with time-lapse fluorescence microscopy, we noted that PI dye in the culture medium entered NCI-H226 cells as the cells swelled, indicating that the integrity of the cell membrane had been destroyed (Supplementary Movie. 1). Additionally, annexin V/7-AAD double staining positive signal has also been detected after the challenge of ORFV (Supplementary Fig. 2c). We further found the release of lactate dehydrogenase (LDH) into tumor cell supernatants, which confirmed that membrane rupture occurred (Fig. 1f). Notably, ORFV-induced cell death correlated with the release of immune stimulant High Mobility Group Box 1 (HMGB1) into the tumor cell supernatant, further supporting that ORFV-triggered pyroptotic cell death is pro-inflammatory (Fig. 1g and Supplementary Fig. 2d). The above observations strongly suggest that oncolytic ORFV was able to trigger tumor cell pro-inflammatory pyroptosis.

**Fig. 2:**
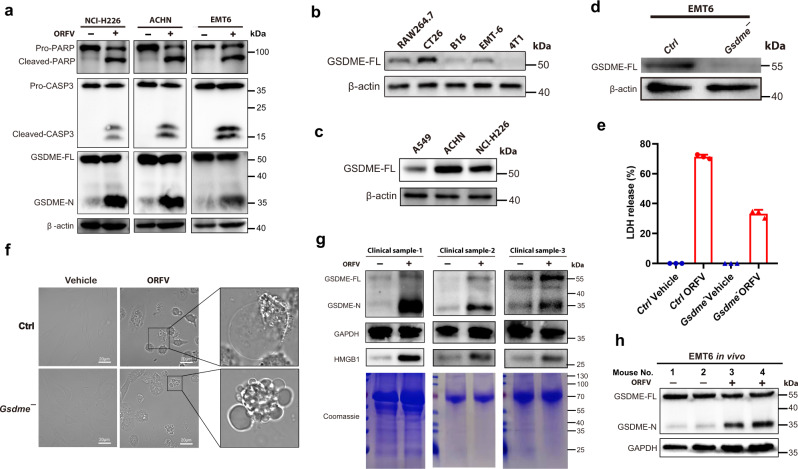
GSDME mediates ORFV-triggered pyroptosis in tumors. **a** Immunoblots for the detection of PARP, GSDME-FL, GSDME-N, and cleaved caspase 3 in ORFV or vehicle-treated (12 h) NCI-H226, ACHN, and EMT6 tumor cell lysates. **b** Immunoblots for GSDME-FL detection in several murine tumor cell lines. **c** Immunoblots for GSDME-FL detection in three human tumor cell lines. **d** Immunoblots for the detection of GSDME protein levels in Ctrl and GSDME-depleted EMT6 cells. **e** LDH release assays were performed in Ctrl or GSDME depletion EMT6 cells after ORFV (MOI = 1) treatment for 16 h (*n* = 3). **f** Images acquired by microscopy display changes in EMT6 cell morphology upon ORFV challenge (MOI = 1) with or without GSDME depletion (scale bar: 20 μm). **g** Immunoblots for the detection of GSDME-FL, GSDME-N, and HMGB1 in ORFV or vehicle-treated human patient tumor samples for 2 days. GAPDH staining was used as the control for GSDME. Coomassie staining is shown as the control for HMGB1. **h** Immunoblots for the detection of GSDME-FL and GSDME-N in ORFV or vehicle-treated (i.t.) EMT6-bearing mice for 2 days. The above experiments were successfully repeated two to three times, and the results are presented as the mean ± SD.

To further clarify the critical factors that contribute to ORFV-induced cell pyroptosis, we and others have treated different tumor cell lines with ORFV and found the activation of caspase 3^27^ (Fig. 2a and Supplementary Fig. 2e). Importantly, activated caspase 3 has recently been confirmed to cleave GSDME, release pore-forming GSDME N-terminus and further induce cell pyroptosis in GSDME-expressing cells, rather than long considered cell apoptosis^16,27^. The above findings inspired us to explore the relationship between ORFV-induced pyroptosis and the caspase 3/GSDME axis. Indeed, under the treatment of ORFV, caspase 3 activation was accompanied by the cleavage of GSDME and the release of GSDME N-terminus in GSDME-expressing cells (Fig. 2a, b, c and Supplementary Fig. 2e). To further confirm the possible causal relationship between GSDME expression and ORFV-induced cell pyroptosis, we constructed GSDME deficient EMT6 cells by harnessing CRISPR/Cas9-based gene modification method, and performed the treatment with ORFV (MOI = 1). We observed that ORFV-triggered pyroptotic cell swelling and LDH release were highly impaired upon the depletion of GSDME, as compared to WT cells (Fig. 2d–f). Loss of GSDME has also switched pyroptotic EMT6 cells (annexin V/7-AAD double positive) into apoptotic cells (annexin V single positive) (Supplementary Fig. 2f).

In agreement with the data generated from tumor cell lines, we investigated the effect of ORFV on ex vivo human colon tumor tissues, and found that GSDME from human colon tumor tissues can be cleaved after the treatment of ORFV for 48 h, which was accompanied by the release of HMGB1 into the supernatant (Fig. 2g). Similarly, we observed GSDME cleavage in EMT6 tumors after 2 days of ORFV (10^5^ TCID_50_) treatment (Fig. 2h).

Given that wild-type (WT) ORFV can trigger GSDME-mediated tumor cell pyroptosis, we further generated ORFV recombinants by deleting a different set of virulence genes, and examine the ability of ORFV recombinants on inducing pro-inflammatory cell pyroptosis. Although one gene deletion recombinant has been reported to be effective in an antitumor study^27^, deletion of dual-gene or even large genome fragments (including several potential virulence genes) still needs to be explored. We previously established OV-SY17Δ120 (hereafter referred to as ORFV-∆120-EGFP), in which the virulence gene *ORFV120* was replaced with *EGFP*^28^. Here, we further designed and established *ORFV120*-*ORFV121* dual-gene deletion recombinant (hereafter referred to as ORFV-∆120-121-EGFP) (Supplementary Fig. 3a). The above operations not only demonstrate that multi-gene deletion in parapoxvirus genome is feasible practically, but also provide potential capacity for accommodating large foreign DNA fragments. Importantly, HMGB1 release and GSDME cleavage were observed upon the challenge of these ORFV recombinants, further confirmed the ability of these ORFV recombinants on triggering tumor cell pyroptosis (Supplementary Fig. 3b).

Collectively, the above data suggest that WT ORFV and ORFV recombinants can trigger GSDME-mediated pyroptosis and cause a subsequent release of pro-inflammatory mediators in GSDME-expressing tumor cells.

### Oncolytic ORFV pre-stabilizes GSDME by decreasing ubiquitination on GSDME and further triggers pyroptosis in GSDME-low tumors

**Fig. 3:**
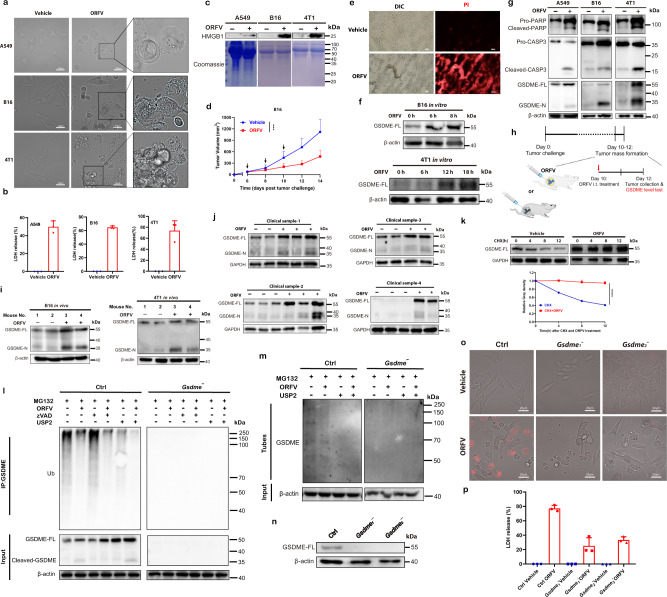
ORFV pre-stabilizes GSDME through decreasing ubiquitination on GSDME and further triggers pyroptosis in GSDME-low tumor cells. **a** Images display changes in cell morphology after ORFV treatment (MOI = 1) for 20, 26, and 30 h, respectively (scale bar: 20 μm). **b** LDH release assays were performed after ORFV treatment (*n* = 3). **c** HMGB1 level detection in supernatants from ORFV-treated A549 (10 h), B16 (12 h), and 4T1 (20 h) cells. **d** Male C57BL/6 mice (*n* = 7) were s.c. engrafted with B16 cells and i.t. treated with ORFV (*P* = 0.0006). **e** Images display PI uptake in B16 tumor tissues after the challenge of ORFV for 2 days (scale bar: 50 μm). Mice were intravenously (i.v.) administrated with PI dye 2 h before tumor collection. **f** GSDME-FL detection in ORFV-treated tumor cell lysates. **g** Detection of PARP, GSDME, and cleaved caspase 3 in ORFV-treated cell lysates for 30 h. **h** Schematic illustration of experimental design. Tumor tissues were collected for GSDME detection 2 days after ORFV intratumorally (i.t.) administration. **i** Detection of GSDME-FL and GSDME-N in ORFV-treated tumor tissue lysates. **j** Detection of GSDME in ORFV or vehicle-treated human patient samples for 20 h. **k** Detection of GSDME stability. Vehicle or ORFV-treated B16 cells were incubated with 100 μM cycloheximide before cell collection. Relative Gray density analysis is at the bottom (*n* = 3) (*P* < 0.0001). **l** Immunoprecipitation analysis of GSDME ubiquitination in Ctrl and GSDME-depleted B16 cells upon ORFV, zVAD, and USP2 treatment for 12 h in the presence of 10 μM MG132. **m** Ubiquitinated GSDME was identified after pulldown with TUBE agarose beads to trap polyubiquitinated proteins from Ctrl and GSDME-depleted B16 cells upon ORFV and USP2 treatment. **n** Detection of GSDME protein levels in Ctrl and GSDME-depleted B16 cells. **o** Images display changes of B16 cell morphology and PI uptake upon ORFV challenge with or without GSDME depletion (scale bar: 20 μm). **p** LDH release assays were performed in Ctrl or GSDME-depleted B16 cells after ORFV treatment for 24 h (*n* = 3). The above experiments were successfully repeated two to three times. ***P < 0.001 and ****P < 0.0001. Two-tailed unpaired Student’s *t*-tests were performed for the statistical analyses in (**d**), and the results are presented as the mean ± SD.

A pivotal study reported that caspase 3/GSDME-mediated pyroptosis is highly dependent on the basal level of GSDME and that tumor cells lacking ‘sufficient’ GSDME develop apoptosis instead of pyroptosis in the context of chemotherapy^16^. Here we asked whether ORFV can trigger inflammatory pyroptosis or noninflammatory apoptosis in GSDME-low tumor cells. To address the question, we first performed the treatment of etoposide or ORFV on GSDME-high cells (EMT6 and CT26) as controls, ORFV-induced pyroptotic cell death was identical to that induced by etoposide, which was indicated by similar PI uptake signals (Fig. 2b and Supplementary Fig. 4a). In contrast, the effects in GSDME-low tumor cells (B16 and 4T1) varied greatly between the two treatments. Etoposide stimulated only mild (B16) or no (4T1) pyroptosis (Supplementary Fig. 4b), while ORFV triggered marked pyroptosis in both of these two cell lines, which was indicated by the morphology of cell membrane bubbles blowing, LDH/HMGB1 release and PI uptake signal (Fig. 3a–c and Supplementary Fig. 4b). In line with the above cellular effect, we found the inhibitory effect of ORFV on B16 and 4T1 tumors (Fig. 3d and Supplementary Fig. 4c, d). To further explore ORFV-induced pro-inflammatory effect in GSDME-low tumors in vivo, we collected B16 tumors from ORFV (10^5^ TCID_50_)-treated mice, and observed that ORFV-induced tumor suppression was accompanied by the appearance of PI-positive cells and the enrichment of cell killing-related genes (Fig. 3e and Supplementary Fig. 5).

Of note, the period of ORFV-triggered pyroptotic cell death in B16 and 4T1 cells was delayed compared with that in CT26 and EMT6 cells (Supplementary Fig. 4a, b). This finding inspired us to explore the change of GSDME protein level in GSDME-low tumors upon the ORFV challenge. Notably, GSDME protein levels in tumor cells increased upon ORFV (MOI = 1) treatment at indicated time points (6 hpi (hours post inoculation) for B16 cell and A549; 12 hpi for 4T1) (Fig. 3f and Supplementary Fig. 6a). Subsequently, the elevated GSDME was further cleaved and 35 kDa of GSDME-N fragments were released (Fig. 3g). Consistently, GSDME protein enhancement and cleavage has been simultaneously observed in the tumors from tumor-bearing mice after intratumoral treatment of ORFV (10^5^ TCID_50_) (Fig. 3h, i and Supplementary Fig. 6b). Importantly, by treating patient primary tumor cells ex vivo with ORFV for 20 h, we have also observed GSDME enhancement and cleavage in these tumors (Fig. 3j).

Next, we further explored the underlying mechanism of GSDME enhancement upon the ORFV challenge. This increase in the GSDME protein level was independent of transcriptional regulation, as no significant changes in *Gsdme* mRNA levels were detected (Supplementary Fig. 6c, d). The above data suggested that GSDME protein stability was enhanced in this context. To examine this hypothesis, cycloheximide (CHX) was first performed to block protein synthesis with or without ORFV. We found that the GSDME level decreased gradually after CHX treatment as expected, while ORFV treatment maintained the GSDME protein level (Fig. 3k). To elucidate how ORFV enhanced GSDME, B16 cell extracts (treated with vehicle or ORFV) were immunoprecipitated with anti-GSDME specific antibody, then ubiquitinated modifications were detected in the anti-GSDME immunoprecipitates. We observed that ubiquitinated GSDME significantly decreased upon ORFV treatment (Fig. 3l). Meanwhile, similar results were obtained in A549 cells after ORFV treatment (Supplementary Fig. 6e). The same cell extracts (treated with vehicle or ORFV) were further immunoprecipitated with Tandem Ubiquitin Binding Entities (TUBEs) reagent-TUBE2, which was followed by western blotting with anti-GSDME antibody. The results showed that ubiquitinated GSDME can be detected in TUBE2 immunoprecipitates in the control cell lysates. When the ORFV challenge was performed, ubiquitinated GSDME from TUBE2 immunoprecipitates dramatically decreased (Fig. 3m). While in GSDME^**−/**−^ cell lysates, the GSDME bands were absent. The above data further support the finding that ORFV treatment enhanced GSDME protein levels.

To better evaluate the potential role of elevated GSDME in the process of ORFV-induced cell pyroptosis, we constructed GSDME deficient B16, B16-F10, and 4T1 cells through CRISPR/Cas9 technique, which was followed by the treatment of ORFV (MOI = 1) (Fig. 3n, and Supplementary Figs. 7a, 8a). We found that ORFV-triggered pyroptotic cell swelling, PI uptake, and LDH release were highly impaired upon the depletion of GSDME, as compared to WT cells (Fig. 3o, p and Supplementary Figs. 7b, c, 8b, c). Additionally, the loss of GSDME has also switched pyroptotic B16 cells (annexin V/7-AAD double positive) into apoptotic cells (annexin V single positive) (Supplementary Fig. 7d).

The above evidences highlight, even in GSDME-low cells, a dual role of oncolytic ORFV in prestabilizing GSDME protein by affecting ubiquitin-dependent GSDME degradation, and in subsequently triggering the cleavage of elevated GSDME and cell pyroptosis.

### Loss of GSDME impairs ORFV-induced antitumor immunity and tumor suppression

**Fig. 4:**
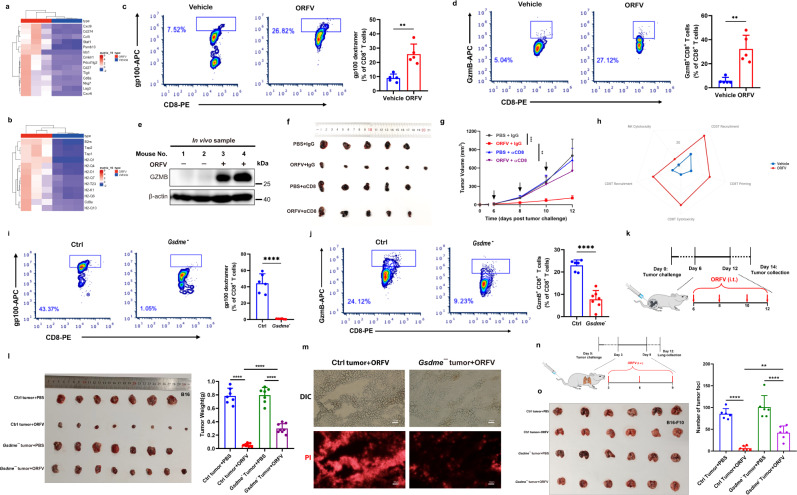
GSDME depletion impairs ORFV-induced antitumor immunity. **a**, **b** Representative heatmap of ORFV-regulated genes based on NanoString PanCancer immune profiling analysis (26) (**a**) and the antigen presentation (**b**). **c** FACS analysis for the detection of melanoma antigen gp100-specific CD8^**+**^ T cells in vehicle or ORFV-treated tumors. Quantitative analysis results are shown on the right (*n* = 5)(*P* = 0.0011). **d** FACS analysis for the detection of GzmB^+^ CD8^**+**^ T cells in vehicle or ORFV-treated tumors. Quantitative analysis results are shown on the right (*n* = 5)(*P* = 0.0010). **e** GzmB detection in vehicle or ORFV-treated B16 tumor lysates. **f**, **g** Male C57BL/6 mice were s.c. engrafted with B16 cells, then i.t. treated with vehicle, ORFV, an anti-CD8 blocking antibody or ORFV, and the antibody (*n* = 5–6). Tumor volumes were shown in (**f**) and (**g**), respectively (***P = 0.0001 and **P = 0.0099). **h** Spider web plot showing the changes of immune cells in the tumor microenvironment after the ORFV challenge. Values represent the percentage of the indicated cells, and the data are the same as those shown for FACS analyses. **i**, **j** Mice were engrafted with Ctrl or *Gsdme*^***-***^ B16 tumor cells, and dextramer detection of melanoma antigen gp100-specific CD8^**+**^ T cells (**i**) (*n* = 6) (*P* < 0.0001) and GzmB^**+**^CD8^**+**^ cytotoxic T cell (**j**) (*n* = 6–7) (*P* < 0.0001) in vehicle or ORFV-treated tumors. **k** Schematic illustration of experimental design. Tumors were collected after ORFV treatment. **l** Ctrl and *Gsdme*^***-***^ B16 tumors were collected after PBS or ORFV treatment, photographs and weights of Ctrl B16 and *Gsdme*^***-***^ B16 tumors were shown (*n* = 7–8) (*P* < 0.0001). **m** PI uptake in Ctrl and *Gsdme*^***-***^ B16 tumors after ORFV challenge (scale bar: 50 μm). **n** Schematic illustration of experimental design. Tumor-bearing mice were i.v. treated with ORFV. Lungs were collected for the image and metastatic foci statistics. **o** Mice i.v. engrafted with Ctrl or *Gsdme*^***-***^ B16-F10 cells were i.v. treated with vehicle or ORFV (*n* = 6). The number of metastatic tumors in the lungs were counted (**P = 0.0084 and ****P < 0.0001). The above experiments were successfully repeated two to three times. *P < 0.05, **P < 0.01, ***P < 0.001, and ****P < 0.0001. Two-tailed unpaired Student’s *t*-tests were performed for the statistical analyses in (**c**, **d**, **i**, **j**), and the results are presented as the mean ± SD. One-way ANOVA with Tukey’s test was performed in (**g**, **l**, **o**), and the results are presented as the mean ± SD. IgG control isotype antibody, αCD8 anti-CD8 blocking antibody.

Given the dual role of oncolytic ORFV mentioned above, we further explored the functional consequence of elevated GSDME in oncolytic ORFV-treated immunologically ‘cold’ tumors. The effect of ORFV on the B16 tumor immune microenvironment was explored using RNA sequencing. The enrichment of pro-inflammatory signaling pathways were revealed in ORFV-treated tumors versus vehicle-treated tumors (Supplementary Fig. 9). Of note, the expression of a group of genes representing T cell-inflamed gene expression profiles (GEPs) (based on NanoString PanCancer immune profiling) were found to be increased upon ORFV challenge^29^ (Fig. 4a and Supplementary Fig. 10a). Meanwhile, a series of antigen presentation-related genes were also dramatically upregulated after ORFV treatment (Fig. 4b). The above data indicates activation of T cell-mediated immune response and suggests a tumor immune microenvironment switch from immunologically “cold” to “hot”. Next, a flow cytometry assay was performed to analyze the infiltrated immune cells in tumors upon the viral challenge. Compared with vehicle-treated controls, we found that infiltrating CD3^+^ and CD8^+^ T cells were increased in B16 tumor tissues treated with ORFV (Supplementary Fig. 10b). Furthermore, the frequency of melanoma antigen-specific CD8^+^ T cells (gp100^**+**^CD8^**+**^ T) was found to be increased, demonstrating that tumor-specific immunity was enhanced (Fig. 4c). Additionally, the percentage of tumor-infiltrating GzmB^+^CD8^+^ T cells among CD8^+^ T cells was significantly increased, suggesting that CTLs functioned to kill tumor cells (Fig. 4d). In line with the above data, GzmB in B16 tumor tissues was upregulated at both the mRNA and protein levels under the same condition (Fig. 4e and Supplementary Fig. 10c). To evaluate the functional contribution of tumor-infiltrating CD8^+^ T cells in the context of ORFV treatment, we depleted CD8^+^ T cells by administrating anti-CD8 blocking antibodies to B16 tumor-bearing mice, and observed that depletion of CD8^+^ T cells significantly reversed ORFV-induced tumor remission (Fig. 4f, g). Additionally, NK cell activation has also been observed upon ORFV treatment, which was in line with the observation from others^9,25^, suggesting that NK cells may also contribute to immune microenvironment remodeling (Supplementary Fig. 10b). The above data demonstrate that oncolytic ORFV is able to reshape the tumor immune microenvironment by recruiting and activating TILs (Fig. 4h).

Given that endogenous GSDME-triggered spontaneous tumor cell pyroptosis in vivo was accompanied by infiltrating cytotoxic lymphocytes^19^, we further dissect the influence of elevated GSDME on activated tumor immune response in immunologically ‘cold’ B16 tumors in the context of ORFV treatment. To investigate this, GSDME KO and WT B16 tumor cells were engrafted into the mice, and this was followed by the treatment with ORFV (10^5^ TCID_50_). We observed that when the GSDME was depleted in the tumors, ORFV treatment was not able to recruit enough tumor-infiltrating CD8^+^, antigen-specific CD8^+^, and GzmB^+^CD8^+^ T cells, as compared to that in WT tumors (Fig. 4i, j and Supplementary Fig. 11). Accompanied by the decrease of tumor-infiltrating lymphocytes, GSDME depletion also contributed to accelerate the tumor growth under the treatment of ORFV, which was confirmed by the changes of endpoint tumor weight (Fig. 4k, l). Furthermore, the PI signal in ORFV (10^5^ TCID_50_)-treated GSDME KO tumors was found much weaker than that in WT tumors, further indicating that PI-positive lytic cell death is GSDME-mediated pyroptosis (Fig. 4m). Additionally, GSDME depletion has also increased the number of metastatic tumors in lung tissues from B16-F10 cell-engrafted mice under the treatment of ORFV (Fig. 4n, o).

Collectively, the data confirmed that oncolytic ORFV-elevated GSDME plays a critical role in the process of ORFV-reshaped tumor immune microenvironment and ORFV-induced tumor suppression in immunologically “cold” tumors.

### ORFV recombinants target, replicate, and cause pyroptosis in tumor

**Fig. 5:**
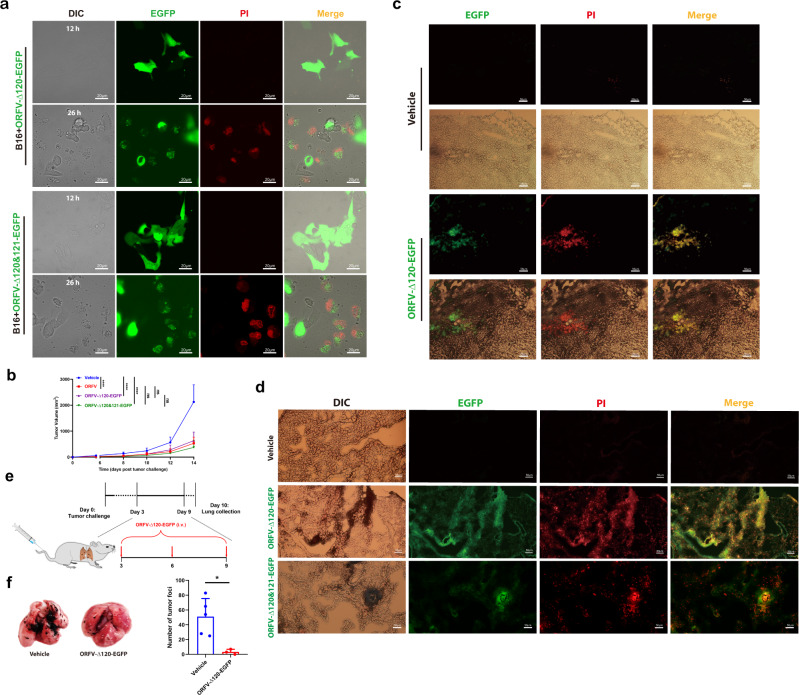
ORFV recombinants trigger, replicate, and cause pyroptosis in tumor cells. **a** Images acquired with a fluorescence microscope display the replication of ORFV-∆120-EGFP (MOI = 1) and ORFV-∆120-121-EGFP (MOI = 1) in B16 cells for 12 h through the EGFP signal. Pyroptotic cell death were displayed through PI uptake signal for 26 h (scale bar: 20 μm). **b** Six weeks old male C57BL/6 mice were s.c. engrafted with B16 cells and i.t. treated with vehicle, ORFV (1 × 10^5^ TCID_50_/mouse), ORFV-∆120-EGFP (1 × 10^5^ TCID_50_/mouse) and ORFV-∆120-121-EGFP (1 × 10^5^ TCID_50_/mouse) (*n* = 7–8). Tumor volume were measured as indicated time points (*P* < 0.0001, *P* < 0.0001, *P* < 0.0001*, P* = 0.4428, *P* = 0.6302, and *P* = 0.2260). **c** Images acquired with a fluores**c**ence microscope display ORFV-∆120-EGFP replication and PI uptake in in situ B16 tumor tissues (scale bar: 50 μm). **d** Images acquired with a fluorescence microscope display the replication of ORFV recombinants (ORFV-∆120-EGFP and ORFV-∆120-121-EGFP) and PI uptake in metastatic tumor cells in the lungs (scale bar: 50 μm). **e** Schematic illustration of experimental design. Tumor-bearing mice were tail intravenous injection (i.v.) treated with ORFV-∆120-EGFP. Lungs were collected for the image. **f** Six weeks old male C57BL/6 mice were i.v. engrafted with B16-F10 cells (1 × 10^5^ cells/mouse) and i.v. treated with ORFV-∆120-EGFP (1 × 10^4^ TCID_50_) (*n* = 3–5). Photographs of lungs and metastatic lesions were counted (*P* = 0.018). The above experiments were successfully repeated two to three times. ns not significant; ****P < 0.0001. One-way ANOVA with Tukey’s test was performed in (**b**). Two-tailed unpaired Student’s *t*-tests were performed for the statistical analyses in (**f**), and the results are presented as the mean ± SD.

Replication-competent oncolytic viruses have been believed to be a promising antitumor strategy, and orthopoxvirus subfamily member vaccinia virus has been shown to target and replicate in human tumor tissues^30^. Here we sought to determine whether or not our established parapoxvirus recombinants were able to work ideally in the same way in vitro and in vivo. ORFV recombinants are armed parapoxvirus carrying *EGFP* transgene for imaging viral replication and distribution (Supplementary Fig. 3a). When B16 tumor cells were firstly infected with different ORFV recombinants (MOI = 1), green fluorescence signals were observed ~12 hpi, indicating that these virus recombinants could replicate in these cells (Fig. 5a). Around another 14 h later, PI dye from the culture medium entered these EGFP-positive tumor cells, leading to the appearance of red fluorescence. Meanwhile, EGFP proteins leaked from ORFV-challenged cells into the cell culture medium and the green fluorescence strength from the cells became weaker, further supporting that viral replication was followed by cell pyroptosis characterized by pore-forming (Fig. 5a and Supplementary Movie. 2).

We next performed an ORFV-based oncolytic study in vivo. B16 tumor-bearing mice were intratumorally treated with vehicle, WT ORFV (10^5^ TCID_50_), ORFV-∆120-EGFP (10^5^ TCID_50_), and ORFV-∆120-121-EGFP (10^5^ TCID_50_), and we found comparable antitumor effect between these viral treatments (Fig. 5b). Given that WT ORFV was able to induce pyroptosis in in situ tumors, we next determined whether or not in situ tumor pyroptosis can be found after the replication viral recombinants. By administrating ORFV-∆120-EGFP recombinants intratumorally into the mice for 2 days, green fluorescence signals in tumor tissues were observed, indicating viral replication in vivo. When PI dye was administered intravenously before tissue collection, red fluorescence (representing PI uptake) appeared in the same regions as the green signal within tumor tissue, suggesting that cell pyroptosis occurred in ORFV-replicating tumor cells in vivo (Fig. 5c). Additionally, we also observed similar effects in ORFV-∆120-EGFP-treated 4T1 tumors (Supplementary Fig. 12).

Then, we mainly focused on the selectivity of different ORFV recombinants for tumor tissue after intravenous infusion in tumor-bearing mice. Malignant murine melanoma B16-F10 cells were intravenously injected into the mice to mimic the establishment of metastatic solid tumors in the lungs. Tumor-targeting effect of ORFV recombinants was examined as indicated in the schematic design (Supplementary Fig. 3a). We found that high-intensity green fluorescence signals appeared only in the tumors (black region) whereas no signal in normal tissues, suggesting that ORFV recombinants were able to selectively enter and replicate in metastatic tumor tissues in the lungs (Fig. 5d and Supplementary Fig. 13). Furthermore, PI signal also appeared in the green signal-positive region, indicating that ORFV recombinants-replicated metastatic tumor cells could undergo lytic cell death-pyroptosis in vivo (Fig. 5d). In support of this, the administration of ORFV-∆120-EGFP (10^4^ TCID_50_) was able to suppress the number of metastatic tumor masses in the lungs (Fig. 5e, f). Together, the above data demonstrate that intravenous infusion of ORFV recombinants undergo blood-borne systemic spread to metastatic tumors, replicate selectively in the tumor cells, and trigger pyroptotic tumor cell death.

### ORFV or ORFV/chemotherapy “dual-therapy” sensitize immunologically “cold” tumors to checkpoint blockade

**Fig. 6:**
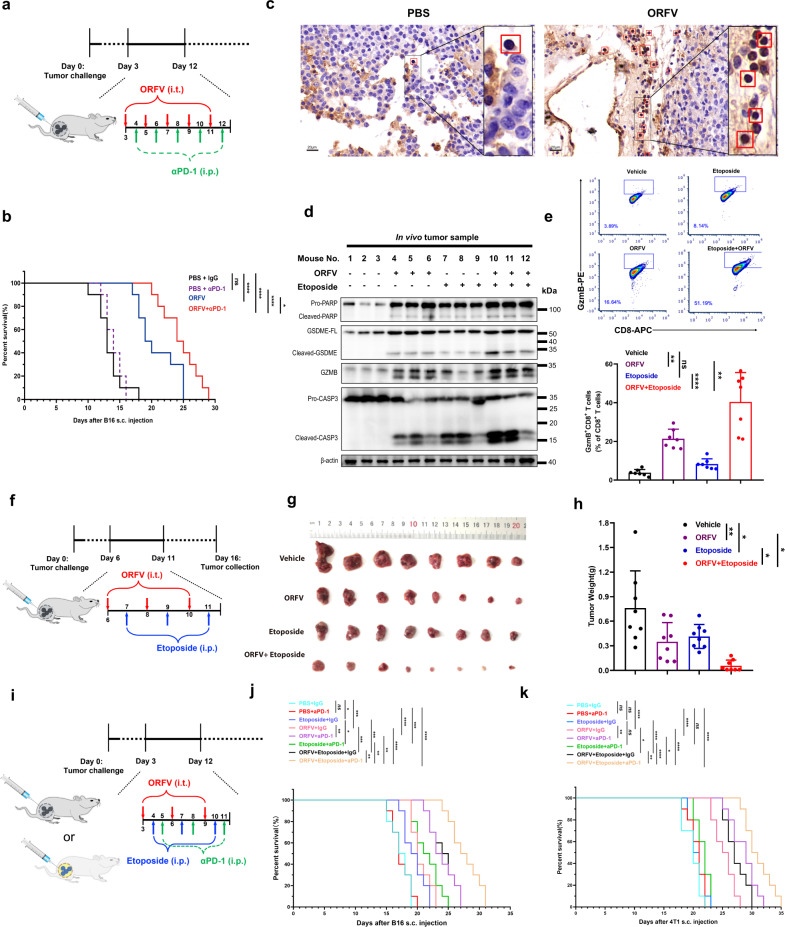
ORFV or ORFV/chemotherapy sensitize immunologically “cold” tumors to PD-1 blockade. **a** Schematic design. Six weeks old male C57BL/6 mice were s.c. engrafted with B16 cells (1 × 10^6^ cells/mouse). B16 tumor-bearing mice (6 weeks old Male C57BL/6) were treated with vehicle, ORFV (1 × 10^5^ TCID_50_/mouse), an anti-PD-1 antibody (5 mg/kg) or combined ORFV and anti-PD-1 as indicated. **b** Kaplan–Meier survival curves are drawn (*n* = 10). **c** Immunohistochemical analysis of PD-1 using paraffin-embedded tumor tissues (scale bar: 20 μm). **d** Six weeks old male C57BL/6 mice were s.c. engrafted with B16 cells (1 × 10^6^ cells/mouse). When the tumors reached 400 mm^3^, the tumors were i.t. treated with ORFV (1 × 10^5^ TCID_50_/mouse) for 2 days, tumor tissues were then collected for immunoblotting assay. Immunoblots for the detection of PARP, GSDME-FL, GSDME-N, caspase 3 cleaved, and GzmB in vehicle, etoposide (5 mg/kg), ORFV (1 × 10^5^ TCID_50_/mouse) and combination-treated tumor lysates. **e** FACS analysis for GzmB^**+**^CD8^**+**^ cytotoxic T cell detection in vehicle, etoposide (5 mg/kg), ORFV (1 × 10^5^ TCID_50_/mouse), and combination-treated tumor tissues (*n* = 7) (*P* = 0.0026, *P* = 0.7384, *P* < 0.0001, and *P* = 0.0011). **f** Schematic design. B16 tumor-bearing mice (6 weeks old male C57BL/6) were treated with vehicle, ORFV (1 × 10^5^ TCID_50_/mouse) and/or etoposide (5 mg/kg) as indicated, which was followed by tumors collection. **g** Images of tumors from different treatments as indicated (*n* = 8). **h** Quantitative analysis of tumor weight. **i** Schematic design. B16 (6 weeks old male C57BL/6) or 4T1 (6 weeks old female BALB/c) tumor-bearing mice were treated with processing as shown in the figure (*n* = 10) (*P* = 0.0049, *P* = 0.0154, *P* = 0.0132, and *P* = 0.0385). **j**, **k** Six weeks old male C57BL/6 mice were s.c. engrafted with B16 cells (1 × 10^6^ cells/mouse). Six weeks old female BALB/c mice were s.c. engrafted with 4T1 cells (1 × 10^6^ cells/mouse). Kaplan–Meier survival curves are drawn for B16 (**j**) (*n* = 10) and 4T1 (**k**) (*n* = 10). The above experiments were successfully repeated two to three times. ns not significant; *P < 0.05, **P < 0.01, ***P < 0.001, and ****P < 0.0001. One-way ANOVA with Tukey’s test was performed in (**e**, **h**), and the results are presented as the mean ± SD. Log-rank test was performed in (**b**, **j**, **k**). IgG control isotype antibody, αPD-1 anti-PD-1 blocking antibody.

Unlike immunologically “hot” tumors that are infiltrated with sufficient T cells, “cold” tumors often display a poor response to PD-1 blockade therapy^5,6^. Indeed, we have observed that anti-PD-1 antibody monotherapy did not affect the survival of immunologically “cold” tumor-bearing mice, which was in line with previous studies^31^ (Fig. 6a, b). Given the obvious limitation of anti-PD-1 antibody, additional strategies are needed to broaden the application scenario of PD-1 blockade. It was recently reported that GSDME spontaneously activated antitumor immunity by pyroptosis and further switched immunologically “cold” tumors into “hot” tumors^19^. In the present study, we have found that i.t. treatment with ORFV could increase the infiltration of PD-1 positive lymphocytes (Fig. 6c). Then, we explored the efficacy of the combination strategy with PD-1 blockade and ORFV-a strategy to harness GSDME for the treatment of ‘cold’ tumors (Fig. 6a). We observed that PD-1 blockade was able to markedly extend the survival of ORFV pretreated mice (Fig. 6b), which further supported that oncolytic ORFV can harness GSDME, recruit CTLs and further remodel tumor immune microenvironment.

Given that the first step of ORFV infection was to stabilize and accumulate GSDME, we wonder if other therapeutic agents could further enhance the cleavage of elevated GSDME and enhance the efficacy of ORFV. Chemotherapy with etoposide has been reported to trigger GSDME-mediated pyroptosis in GSDME-expressing cells^16^. Inspired by the above findings, we treated B16 tumor-bearing mice with combination therapy, including etoposide and ORFV. After two rounds of ORFV treatment, the GSDME level was increased and cleaved in tumor tissues as expected (Fig. 6d). Notably, GSDME was markedly cleaved when etoposide was administered together with ORFV as compared to ORFV monotherapy, suggesting that a higher level of pyroptotic cell death occurred with combination therapy in vivo (Fig. 6d). Meanwhile, the “dual-therapy”-enhanced level and activity of GSDME were accompanied by the increased proportion of GzmB^+^CD8^+^ T cells as well as the increased GzmB level in tumors, suggesting more CTLs infiltration (Fig. 6d, e). Furthermore, we found that the dual-therapy achieved greater tumor reduction than the corresponding monotherapies (Fig. 6f–h). Given the compelling T cell recruiting capacity of dual-therapy, “triple-treatment” with etoposide, ORFV, and anti-PD-1 antibody was administered at a low frequency to treat B16 cell-engrafted mice (Fig. 6i). We observed that PD-1 blockade extended the survival of low frequency ‘dual-therapy’ pretreated mice (Fig. 6j). Similarly, “triple-treatment” displayed an additive effect on the survival of triple-negative breast cancer 4T1-bearing mice (Fig. 6k). The above in vivo work not only supported the point of view that ORFV or ORFV/chemotherapy “dual-therapy”-induced pyroptosis can recruit CTLs to create an inflamed tumor microenvironment, but also suggested a way to make PD-1 blockade therapy applicable for killing immunologically “cold” tumors.

## Discussion

**Fig. 7:**
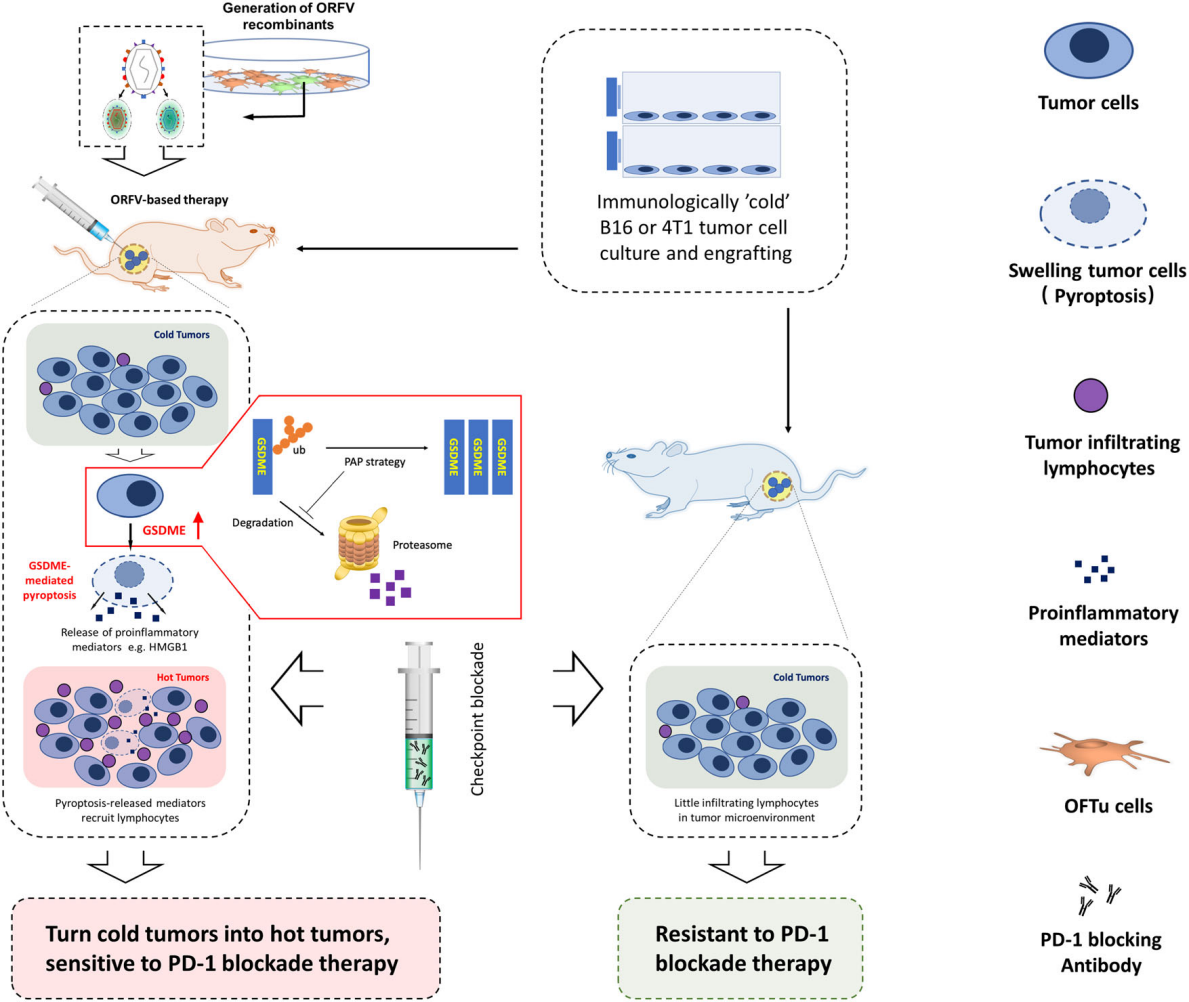
ORFV induces GSDME-mediated pyroptosis and activates antitumor immunity. We can generate different ORFV recombinants with one or two genes deletion, which are armed with the *EGFP* gene for imaging. WT ORFV and ORFV recombinants are able to trigger GSDME-mediated pyroptosis in tumor cell lines, in vivo tumor tissues, and ex vivo human colon cancer tissues. ORFV can pre-stabilize GSDME protein levels in GSDME-low tumor cells through decreasing ubiquitination of GSDME, which was followed by GSDME cleavage and pyroptotic cell death. ORFV-triggered GSDME-mediated tumor pyroptosis recruits CTLs into the tumor microenvironment, which is accompanied by the release of inflammatory mediators. This remodels the tumor microenvironment and turns immunologically “cold” tumors into “hot” tumors, thereby sensitizes these tumors to checkpoint blockade. While the immunologically “cold” tumors are not able to respond to checkpoint blockade.

We discovered that an ORFV-based therapeutic strategy can trigger GSDME-mediated tumor cell pyroptosis. Importantly, the GSDME protein level was increased upon ORFV challenge in tumor cell lines, primary human tumor tissues and in vivo murine tumors, which was owing to the decrease of ubiquitination on GSDME. Under the challenge of ORFV, depletion of GSDME blocked pyroptosis, antitumor immunity, and tumor response. ORFV recombinants targeted, replicated, and induced pyroptosis in immunologically “cold” tumor models. Notably, the ORFV-based therapeutic strategy sensitized immunologically noninflamed (cold) tumors to checkpoint blockade (Fig. 7).

Several viruses have been reported to trigger pyroptosis in normal cells. Human immunodeficiency virus (HIV) was shown to induce CARD 8-dependent pyroptosis in CD4^**+**^ T cells^32^, while human respiratory syncytial virus (RSV) induced caspase-1 and ASC-NLRP3 inflammasome-dependent pyroptosis in macrophages^33^. In addition, the influenza A virus and vesicular stomatitis virus were confirmed to trigger GSDME-mediated pyroptosis in epithelial cells and bone marrow-derived macrophages, respectively^34,35^. With regard to the ORFV challenge, we observed the activation of caspase 3/GSDME axis and subsequent pyroptosis induction in not only GSDME-high but also GSDME-low cells. Given that GSDME-low tumors turned to apoptosis instead of pyroptosis upon caspase 3 activation^16^, the contradiction suggested that the GSDME protein has changed after virus infection. Interestingly, we found the level of GSDME protein but not mRNA has increased, and the increase of GSDME protein in B16 cells was observed as fast as 6 hpi (Fig. 3f). Notably, although some tumor cell lines (e.g., 4T1 cells) display low GSDME protein level compared with GSDME-high tumor cells (e.g., CT26 cells)^16^, a basal mRNA level is detectable^19^, suggesting that the GSDME protein is not stable in these cells. Here, we further discovered that the decrease of GSDME ubiquitination was accompanied by an increase in GSDME stability. A pivotal study performed decitabine treatment for 6 days to increase *GSDME* mRNA level for accumulating enough GSDME in GSDME-low tumor cells^16^. In another study, the GSDME protein has been found accumulated in esophageal squamous cell carcinomas after combination treatment with a PLK1 inhibitor and cisplatin for 16 h, making pyroptosis-mediated tumor suppression possible^36^. Importantly, the rapid stabilization and accumulation of GSDME by ORFV-based strategy within hours is impressive and has strong translational prospects in the context of pyroptosis-based antitumor-targeted therapy. Of note, the underlying mechanism of ORFV-induced GSDME enhancement still needs to be explored. It was reported that virus-encoded proteins could modulate cellular fate by interacting with host E3 ubiquitin ligases^37,38^. Therefore, exploring the effect of ORFV on the function and expression of E3 ubiquitin ligases could be one way to study ORFV-triggered GSDME deubiquitination.

ORFV therapy has been reported to display an immunomodulatory capacity^9,39,40^. In cancer therapy, the study of exploring ORFV immunomodulatory potential is mainly focused on its activation of NK cells and the secretion of cytokines^9,40^. From our RNA-seq data, not only the NK cell function-related gene *Nkg7* (natural killer cell granule protein 7) but also a series of T cell-inflamed genes were upregulated upon ORFV challenge (Fig. 4a). The activation and contribution of tumor-infiltrating CTLs and have been confirmed in the process of ORFV-induced tumor regression (Fig. 4c–g). Indeed, similar observations have been found in antitumor therapy using a herpes simplex type 1 OV or seasonal influenza^41–43^. OVs administration in situ behaves like tumor vaccines and releases tumor-associated antigens (TAAs), which can be presented to tumor-infiltrating CD8^+^ T cells and initiate T cell priming^1,5^. In this study, we found that antigen presentation-related genes were greatly upregulated after ORFV treatment (Fig. 4b). Deletion of these genes in tumor cells leads to loss of sensitivity to CD8^+^ T cell-mediated killing^44^. Indeed, we observed an increase in gp100 antigen-specific CD8^+^ T cells in ORFV-challenged B16 tumor tissues, suggesting tumor-specific T cell response was primed (Fig. 4c).

Oncolytic ORFV has potential merits warranting consideration as a future antitumor strategy. Firstly, WT ORFV infection-induced diseases are not serious in humans and display self-limiting properties in terms of clinical symptoms^39,45,46^. Additionally, it was shown that deletion of one potential virulence gene can impair the pathogenicity of ORFV, which provides conditions for developing ORFV as a safer therapeutic platform^28,47–49^. Indeed, studies are trying to accept ORFV-based vectors as a vaccine development strategy for viral and neoplastic diseases^50,51^. Furthermore, based on the ORFV strategy, we were able to even deplete more virulence genes in the viral genome, without changing the tropism, replication, pro-pyroptotic characteristic, and antitumor ability (Fig. 5). This feature makes ORFV a powerful platform to carry larger foreign inserts in the future development. Secondly, although immune responses occur after ORFV infection, effective neutralizing antibodies specific for ORFV have rarely been found^45,52–54^. Additionally, when ORFV was harnessed as a delivering vector, CD8^**+**^ T cells specific for ORFV-derived antigens were not found^45^. This indicates that neither specific humoral nor cellular immunity against ORFV could be stimulated. Thus, ORFV-based therapy can be repeatedly administered to produce a persistent response, which makes this approach superior to other OV strategies, there is no need to switch OV serotype or use another virus for repeated doses^40^. Thirdly, ORFV can be delivered systemically and has a tumor preference, it is due to the enhanced permeability and retention effect in tumors, which was described in other viral vector applications^55,56^. Other possible reasons for the selective replication of ORFV in cancer cells may be impairments in apoptotic pathways, defects in the cell IFN pathway, and excessive activation of epidermal growth factor receptor/Ras signaling in tumor cells^57–59^. Notably, there are still limitations of ORFV-based study. Firstly, viral proteins that affect the oncolytic capacity of ORFV are poorly studied, and the key components still need to be explored. Secondly, it is hard to evaluate the efficacy of ORFV in clinical studies, as clinical data about its antitumor effect is rare. Based on the above issues, we will carry out exploratory work in the future. It is hoped that further in-depth research on this oncolytic virus can solve more problems in clinical application in the future.

Checkpoint blockade is considered an unprecedented breakthrough in cancer therapy, but it is effective in only a limited proportion of patients because many patients have nonresponsive “cold” tumors^60^. It was reported that OV therapy with the Maraba virus sensitizes breast cancers to checkpoint blockade^4^. Here, we show ORFV strategy reshaped the tumor microenvironment and switched ‘cold’ tumors into “hot” and further sensitizes the nonresponsive tumors to PD-1 therapy (Fig. 6b). Interestingly, low-dose of etoposide further enhanced the cleavage of ORFV-elevated GSDME and thereby recruited more cytotoxic lymphocytes. From a translational perspective, we highlighted that the ORFV strategy was able to be an attractive anticancer biotherapeutic for either monotherapy or combinational approaches with checkpoint blockade.

## Methods

### Cell culture and reagents

ACHN (SCSP-5063), NCI-H226 (SCSP-5073), A549 (SCSP-503), 4T1 (SCSP-5056), B16 (TCM 2), B16-F10 (TCM36), CT26 (TCM37), and RAW264.7 (SCSP-5036) tumor cell lines were obtained from National Collection of Authenticated Cell Cultures. EMT6 (CL-0573) purchase from Procell (Wuhan, China). Primary ovine fetal turbinate (OFTu) cells were isolated and cultured in our laboratory. EMT6, CT26, NCI-H226, and 4T1 cells were maintained in RPMI-1640 medium (HyClone) supplemented with 10% FBS, 100 U/mL penicillin, and 100 μg/mL streptomycin. B16, B16-F10, and OFTu cells were maintained in DMEM (HyClone) supplemented with 10% FBS, 100 U/mL penicillin, and 100 μg/mL streptomycin. ACHN cells were maintained in MEM medium (HyClone) supplemented with 10% FBS, 100 U/mL penicillin, and 100 μg/mL streptomycin. A549 cells were maintained in Ham’s F-12K (Kaighn’s) Medium (HyClone) supplemented with 10% FBS, 100 U/mL penicillin, and 100 μg/mL streptomycin. Anti-GSDME (EPR19859, ab215191), anti-HMGB1 (ab18256), anti-GZMB (EPR22645-206, ab255598), recombinant human USP2 protein (ab198439), and Alexa Fluor®647-conjugated anti-melanoma gp100 (EP4863(2), ab246730) were purchased from Abcam. Caspase 3 antibody (9662), anti-ubiquitin (P4D1) Mouse mAb (3936 T), GAPDH (D16H11) XP® Rabbit mAb (5174), PARP (46D11) Rabbit mAb (9532), β-Actin (D6A8) Rabbit mAb (8457), PD-1 (Intracellular Domain) (D7D5W) XP® Rabbit mAb (84651), anti-mouse IgG HRP-linked antibody (7076), and anti-rabbit IgG HRP-linked antibody (7074) were purchased from Cell Signaling Technology (CST). Anti-mouse CD16/32 (S17011E, 156603), FITC-conjugated anti-mouse CD45 (I3/2.3, 147710), APC-conjugated anti-mouse CD3 (17A2, 100236), PE-conjugated anti-mouse CD8a (53-6.7, 100708), APC-conjugated anti-mouse CD8a (53-6.7, 100712), APC anti-human/mouse Granzyme B (QA16A02, 372204), and PE anti-human/mouse Granzyme B Recombinant (QA16A02, 372207) were purchased from BioLegend. InVivoMab anti-mouse PD-1 (CD279) (29 F.1A12, BE0273) and InVivoMab anti-mouse CD8α (2.43, BE0061) were purchased from BioXCell. Etoposide (S1225), MG132 (S2619), Propidium Iodide (S6874), and CHX (S7418) were obtained from Selleck. 7-AAD Viability Staining Solution (00-6993-50) was purchased from eBioscience. Annexin V-FITC (APOAF) was obtained from SIGMA. LDH Cytotoxicity Assay Kit (C0017) was purchased from Beyotime. Tandem Ubiquitin Binding Entities (TUBEs) reagent-Agarose-TUBE2 (UM402) from LifeSensors.

### Human colon tumor samples

Colon tumor samples were collected from patients who underwent tumorectomy at the department of Gastrointestinal Surgery, the First Hospital of Jilin University (Changchun, China). Four patient tumor tissues were randomly collected, two were from male patients, while two were from female patients, and there is no potential self-selection bias. This study was conducted in accordance with the Declaration of Helsinki, and protocols of the ex vivo studies were approved by the Ethics Committee of The First Hospital of Jilin University. Written informed consent was obtained from all individuals. Patient tumor tissues were cut into small pieces and cultured in RPMI-1640 medium (HyClone) supplemented with 10% FBS, 100 U/mL penicillin, and 100 μg/mL streptomycin. All tumor samples were treated with ORFV at indicated time points, which was followed by sample lysing and Western blotting assay.

### ORFV propagation, purification, and observation

ORFV were propagated in OFTu cells. Briefly, OFTu cells at 80% confluence were inoculated with ORFV or ORFV recombinants for 1 h at 37 °C in an FBS-free medium, and then a complete culture medium was added for further culture. Observation of cytopathic effects (CPE) was performed during culture. Cells showing CPE were collected and subjected to three cycles of repeated freeze-thaw cycles. The released viruses were then purified through sucrose gradient ultracentrifugation (Himac CP100WX Preparative Ultracentrifuge) at 4 °C and 50,000 × *g* for 1.5 h. Two microliters of virus liquid were applied to a 200-mesh screen and observed via transmission electron microscopy (HITACHI). The viral titer was determined through TCID_50_ calculation.

### Construction of the ORFV recombinants

The ORFV strain OV-SY17 was used as the WT virus for cell infection and tumor treatment^61^. The OV-SY17∆120 mutant (named ORFV-∆120-EGFP in this work) was designed and modified as described in our previous work^28^. Briefly, a recombination cassette was generated by integrating the VV7.5 promoter sequence, EGFP reporter gene, and ORF120 flanking regions into the PUC57 vector. Then, OFTu cells were transfected with the recombination cassette and infected with ORFV, creating the ORFV-∆120-EGFP recombinant via homologous recombination. For ORFV-∆120-121-EGFP recombinants establishment, a recombination cassette was generated by integrating the VV7.5 promoter sequence, EGFP reporter gene, and ORF120-121 flanking regions into the PUC57 vector.

### In vitro cell pyroptosis determination

Tumor cells were seeded in glass-bottom culture dishes (BIOFIL, BDD-012-035) and treated with virus and drug for the indicated times. For cell morphology examination, bright-field images of swelling cells with bubbles were taken. For the PI uptake assay, PI dye (2 μg/mL) was added to the medium before observation. For both bright-field and fluorescence images of pyroptotic cells, images were captured using a fluorescence microscope (OLYMPUS, FV3000). Photos were displayed and organized by using Adobe Photoshop CS5 and Adobe Illustrator CS5. LDH release assays were performed based on the manufacturer’s instructions (Promega). For the in vitro and in vivo GSDME cleavage assays, tumor cells and tissues were collected after treatments, and western blotting was performed to determine the protein level of full-length GSDME and its N-terminal fragment. Additionally, the amount of HMGB1 protein in the supernatant was determined by western blotting.

### In vivo PI staining assay for tumor cell pyroptosis

Tumor-bearing mice were treated with drug or virus as indicated, followed by i.v. PI administration (2.5 mg/kg) one hour before euthanasia. Tumor tissues or lungs were collected and placed in OCT Embedding Medium (Thermo Fisher Scientific) for freezing. Tumor or lung tissue sections were cut at a thickness of 10 μm, followed by observation using a fluorescence microscope (OLYMPUS, BX53). The GFP signal enriched in tumor tissues was also imaged if the tumors were previously treated with the ORFV-120-EGFP and ORFV-∆120-121-EGFP. Photos were displayed and organized by using Adobe Photoshop CS5 and Adobe Illustrator CS5.

### Immunohistochemical

Immunohistochemical (IHC) staining for PD-1 expression was conducted using sections obtained from the formalin-fixed, paraffin-embedded tumor specimens. The sections were then de-paraffinized, rehydrated, and subjected to antigen retrieval, referring to the instructions of the UltraSensitive SP (Mouse/Rabbit) IHC kit. All images are obtained through the PANNORAMIC MIDI II automatic digital slide scanner (3DHISTECH, Budapest, Hungary). Photos were displayed and organized by using Adobe Photoshop CS5 and Adobe Illustrator CS5.

### RT-qPCR analysis

Total RNA was extracted from tumor tissues and cells with TRIzol (Invitrogen). cDNA was synthesized using PrimeScript reverse transcriptase (TaKaRa). QPCR was performed using CFX96 Touch Real-Time PCR Detection System (Bio-Rad). Relative changes in mRNA expression were calculated using the comparative cycle method (2^−∆∆Ct^). The primer sequences used in the experiment are shown in Supplementary Table 1.

### Flow cytometry assay for TIL detection

Tumor tissues from mice were weighed, minced into small pieces, and ground to make cell suspensions in PBS. A 70-µm cell strainer was used to filter the cells to generate a single-cell suspension. The cells were washed with FACS buffer by centrifugation and resuspended in FACS buffer for further staining. Anti-CD16/CD32 reagent was used for blocking before specific antibody staining. For TIL staining, FITC-conjugated anti-mouse CD45, APC-conjugated anti-mouse CD3, APC/PE-conjugated anti-mouse CD8a, and Alexa Fluor®647-conjugated anti-melanoma gp100 were used to perform staining. For the detection of cytotoxic lymphocytes, cells were first stained for cell surface markers for 30 min, and then the cells were fixed and permeabilized using fixation/permeabilization buffer and stained with PE/APC-conjugated anti-human/mouse GZMB. Raw data of flow cytometry was collected by Beckman Coulter CytoFLEX, which was followed by the analyses using FCS Express 7 software.

### Establishment of knockout cells with CRISPR/Cas9

Two gRNAs targeting *Gsdme* were designed, and the sequences were as follows: 5′-CGGGGCTATTGGGACAGTCG-3′ and 5′-TTTCTGCTAGTGCGCTGACC-3′. The gRNAs were ligated with a BbsI (Thermo Fisher Scientific)-digested PX459 vector. The recombinant plasmids were transfected into EMT6, B16, 4T1, and B16-F10 cells with Lipofectamine 3000 transfection reagent (Thermo Fisher Scientific), and the cells were selected with 2.5 μg/mL puromycin (Sigma Aldrich). Finally, *Gsdme*-knockout cell lines were assessed by western blotting.

### Western blotting

Tumor cells in culture were washed with PBS twice and then lysed in lysis buffer supplemented with 0.01% EDTA (pH 7.5), 150 mM NaCl, 0.1% Triton X-100 and a protease inhibitor cocktail. After protein concentration determination, cell extracts were mixed with SDS loading buffer, followed by SDS-PAGE and transfer to a PVDF membrane. Membranes were blocked with 5% milk in PBST buffer at 4 °C for 2 h before incubation with primary antibodies against GzmB (1:1000, Abcam), GSDME (1:1000, Abcam), PARP (1:1000, CST), Caspase 3 (1:1000, CST), HMGB1 (1:1000, Abcam), Ubiquitin (1:1000, Abcam), GAPDH (1:1000, CST), and β-actin (1:1000, CST) for 2 h at room temperature or at 4 °C overnight. Secondary antibodies were incubated with the PBST-washed membranes for 1 h. After washing with PBST three times, the membranes were incubated with ECL substrate for detection by using Tanon 5200 Chemiluminescent Imaging System. The density of protein bands was quantified by using ImageJ (NIH, 1.50i) software. Uncropped and unprocessed scans of the blots can be found in the Source Data file.

### Co-immunoprecipitation (Co-IP) assay

B16 and A549 cells were treated with vehicle or ORFV (MOI = 1) for 12 h. Cell extracts were immunoprecipitated with anti-GSDME antibody-protein A/G agarose beads (Thermo Fisher) at 4 °C overnight. The beads were washed three times with lysis buffer and suspended in SDS-PAGE. Immunoblotting detection was performed using an anti-Ubiquitin antibody.

### TUBEs pulldown and immunoprecipitation

About 1 × 10^7^ B16 cells were collected and put in 500 μL lysis buffer with 50 μM PR619 and 5 mM o-phenanthroline. About 20 μL TUBEs (UM402) were added in 500 μL cell lysate, and incubated in a 4 °C shaking table for 6 h. Collect TUBEs after 5000 × *g* centrifugation. Wash TUBEs with 1 mL TBS-T, collect by low-speed centrifugation, and aspirate the supernatant leaving a small volume cushion so as to avoid disturbing the beads and repeat twice. For western blotting, TUBEs and SDS loading buffer were mixed and immediately heated to 100 °C for 15 min. Finally, the GSDME ubiquitination in the sample was detected by western blotting.

### Mouse tumor models and treatments

All the mice used in this study were obtained from Liaoning Chang Sheng Biotechnology Co., Ltd (https://www.lncssw.com/product/5/). All animals used in this study were chosen randomly. All animals were kept in a standard laboratory and fed sterile food (Chang Sheng Biotechnology, China) and water. The laboratory was pathogen-free conditions with central air that was controlled by a thermostat (25 °C), relative humidity between 40–60 rH, and provide 12 h light daily. A total of 5 × 10^5^ B16 cells were implanted s.c. in the left flank of 6–8 weeks C57BL/6 male mice, or 5 × 10^5^ 4T1 or EMT6 cells were implanted s.c. in the left flank of 6–8 weeks BALB/c female mice. To establish a lung metastasis model, 5 × 10^5^ B16-F10 cells were intravenously injected into 6–8 weeks C57BL/6 male mice. ORFV (1 × 10^4^ to 1 × 10^5^ TCID_50_) treatment was performed through either i.v. or i.t. administration as described above. Etoposide (5 mg/kg), anti-CD8 blocking antibody (5 mg/kg), and anti-PD-1 blocking antibody (5 mg/kg) treatments were performed with an intraperitoneal (i.p.) injection method based on the experimental timelines shown in the figures. The length (L) and width (W) of each tumor were measured daily with calipers once tumors were visible, and tumor volume was calculated with the following formula (L × W^2^)/2. The tumor volumes of all tumor-bearing mice involved in this study were controlled within 2,500 mm^3^, which is in accordance with the permission from the ethics committee in the College of Veterinary Medicine of Jilin University. The maximal tumor size/burden was not exceeded. Euthanasia of animals, after i.p. 100 mg/kg tribromoethanol, an overdose of thiopental (400 mg/kg, i.p.), and tumors were harvested when the indicated time points or a specified volume was reached. All of the animal experiments were performed in strict accordance with the guidelines set by the Chinese Regulations of Laboratory Animals and Laboratory Animal Requirements regarding Environment and Housing Facilities. The experimental protocol was approved by the ethics committee in the College of Veterinary Medicine of Jilin University.

### Statistics

All animals were randomly grouped, and cell and animal experiments were performed two to three times. Data were analyzed with GraphPad Prism 9. All data were presented as the mean ± SD, unless otherwise stated. Significance is presented as *P < 0.05, **P < 0.01, ***P < 0.001, and ****P < 0.0001. Other details of the statistical tests are described in the individual figure legends. RNA-seq data were analyzed with R (version 4.0.3).

### Reporting summary

Further information on research design is available in the Nature Portfolio Reporting Summary linked to this article.

## Supplementary information

Supplementary Information

Description of Additional Supplementary Files

Supplementary Movie 1

Supplementary Movie 2

Reporting Summary

## Data Availability

The RNA-seq data used in this study are available in the Gene Expression Omnibus (GEO) Database under accession code GSE206634. All the other data generated or analyzed during this study are available from the corresponding author upon reasonable request. Source data are provided with this paper.

## Source data

Source Data
