# Supplementary material for: Oncolytic Parapoxvirus induces Gasdermin E-mediated pyroptosis and activates antitumor immunity
Source: Nat Commun. 2023 Jan 14;14:224. doi: 10.1038/s41467-023-35917-2 (PMC9840172; doi:10.1038/s41467-023-35917-2)
Supplement: Supplementary file 1 — Supplementary Information [file 41467_2023_35917_MOESM1_ESM.pdf]

Oncolytic Parapoxvirus induces Gasdermin E-mediated pyroptosis and activates antitumor immunity

Jing Lin[1,7], Shihui Sun[1,7], Kui Zhao[1,7], Fei Gao[2,7], Renling Wang[1,7], Qi Li[1], Yanlong Zhou[1], Jing Zhang[1], Yue Li[1], Xinyue Wang[1], Le Du[1], Shuai Wang[1], Zi Li[1], Huijun Lu[1], Yungang Lan[1], Deguang Song[1], Wei Guo[3], Yujia Chen[4], Feng Gao[1], Yicheng Zhao[5], Rongrong Fan[6], Jiyu Guan[1*], Wenqi He[1*]

[1] Key Laboratory of Zoonosis, Ministry of Education, College of Veterinary Medicine, Jilin University, 130062 Changchun, China

[2] Department of Laboratory Animals, College of Animal Science, Jilin University, 130062 Changchun, China

[3] Department of Hematology, The first hospital of Jilin University, 130021 Changchun, China

[4] Department of Gastrointestinal Surgery, The first hospital of Jilin University, 130021 Changchun, China

[5] Changchun University of Chinese Medicine, Changchun, China

[6] Department of Biosciences and Nutrition, Karolinska Institutet, 14183 Huddinge, Sweden.

[7] These authors contributed equally: Jing Lin, Shihui Sun, Kui Zhao, Fei Gao, Renling Wang

[*] Correspondence: jiygua@jlu.edu.cn; hewq@jlu.edu.cn

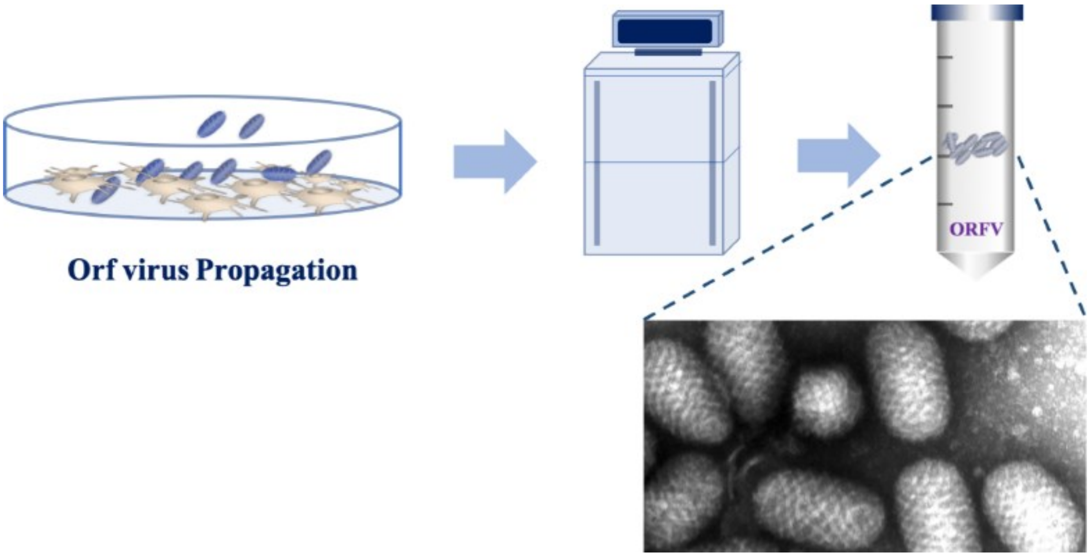

Supplementary Figure 1. The preparation of ORFV. ORFV was propagated in OFTu cells and purified through sucrose gradient ultracentrifugation. Purified ORFV was observed via transmission electron microscope. Viral titer was determined through $TCID_{50}$ calculation.

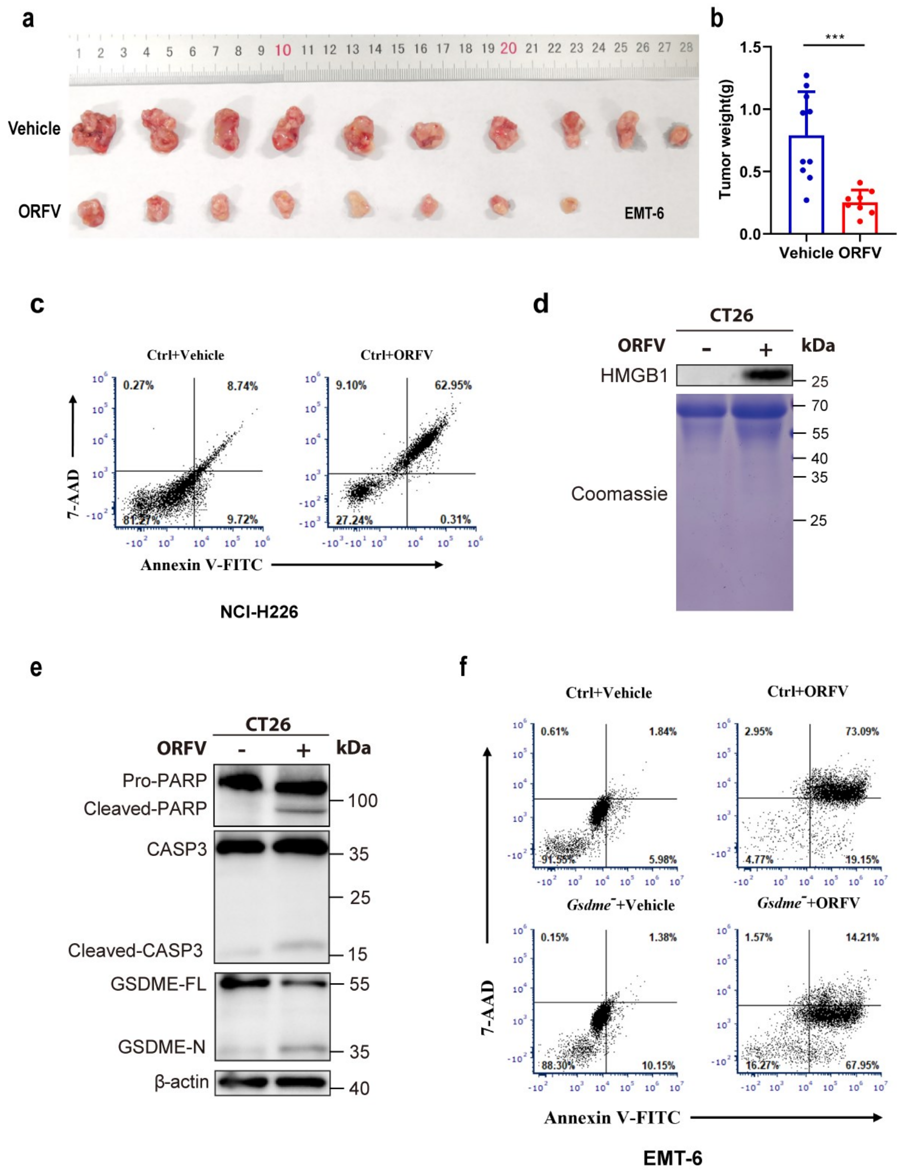

Supplementary Figure 2. ORFV inhibits tumor growth and triggers pyroptosis in GSDME-expressing tumor cells. a-b 6 weeks old female BALB/c mice were s.c. engrafted with EMT6 cells (1×10^6 cells/mouse). EMT6 tumors were collected after ORFV i.t. treatment, photographs (a) and weights (b) were shown (n=8-10) (*P*=0.0001). **c** FACS of 7-AAD and Annexin V- FITC NCI-H226 cells. **d** HMGB1 level detection in the supernatant from ORFV-treated CT26 cells. Coomassie staining was shown as the control. **e** Immunoblots for the detection of PARP, GSDME-FL, GSDME-N as well as caspase 3 cleavage in ORFV-treated CT26 cell lysates. **f** FACS of 7-AAD and Annexin V-FITC with or without GSDME depletion EMT-6 cells. The above experiments were successfully repeated 2-3 times. *** *P*<0.001. Two-tailed unpaired Student's t-tests were performed for the statistical analyses in (b) and the results were presented as means ± SD.

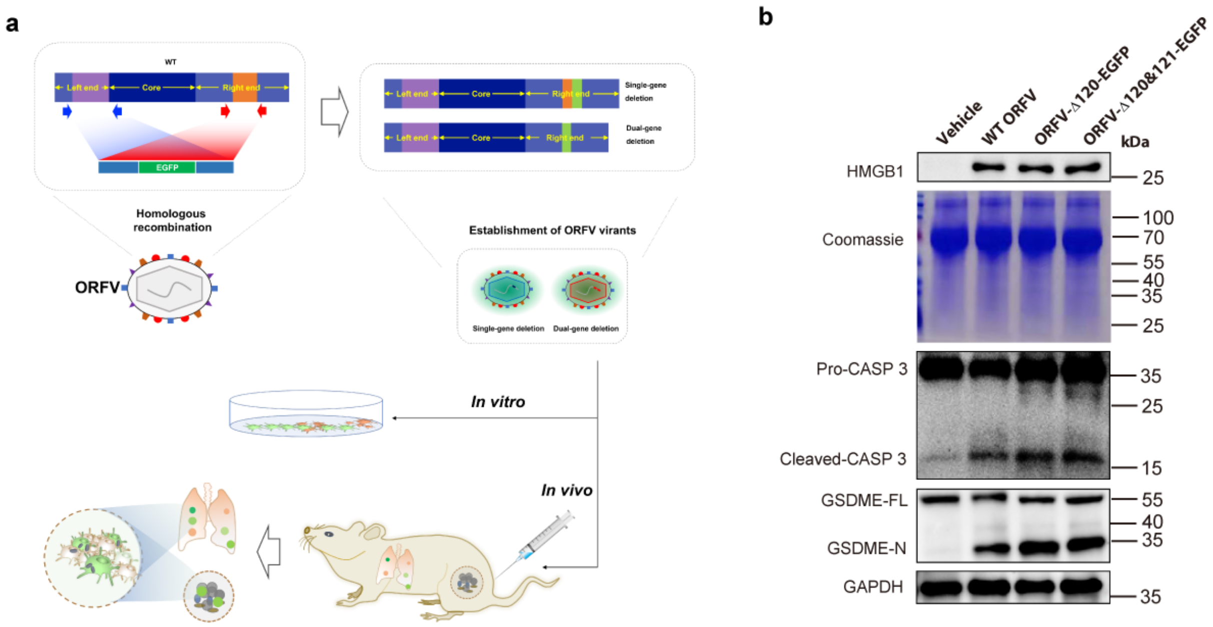

Supplementary Figure 3 ORFV recombinants schematic design a Schematic design showing for constructing the ORFV recombinants through homologous recombination. *ORFV120*, *ORFV120-121* fragments in the ORFV genome were replaced with *EGFP* gene for imaging *in vivo* and *in vitro*. **b** HMGB1 level detection in supernatants from ORFV recombinants or vehicle-treated (20 h) A549 cells. Coomassie staining is shown as the control. Immunoblots for the detection of GSDME-FL, GSDME-N and cleaved caspase 3 in ORFV recombinants or vehicle-treated human A549 cell lysates. The above experiments were successfully repeated 2-3 times.

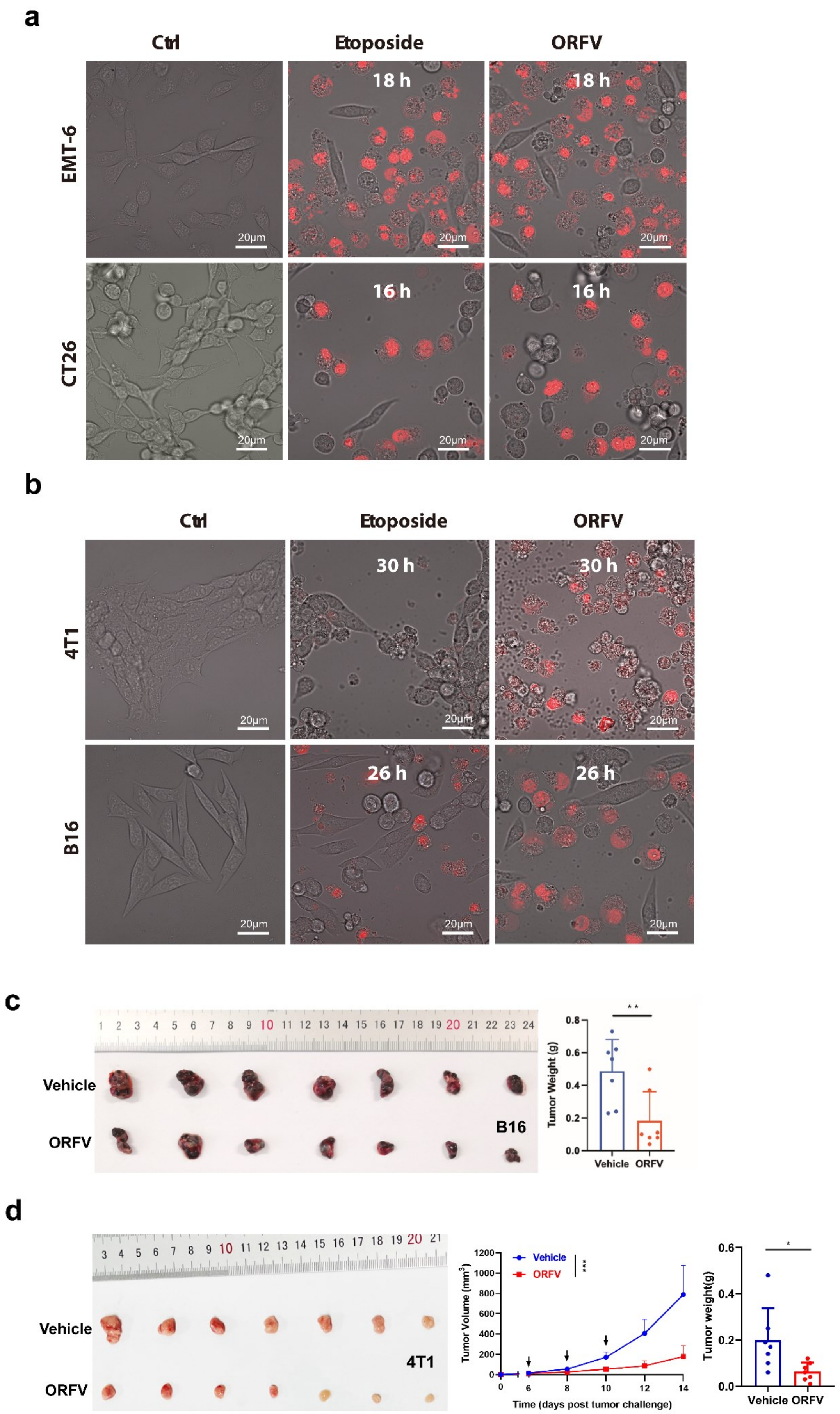

a
Ctrl
Etoposide
ORFV
EMT-6
CT26
18 h
16 h
20μm
b
Ctrl
Etoposide
ORFV
4T1
B16
30 h
26 h
20μm
c
Vehicle
ORFV
B16
Tumor Weight (g)
0.8
0.6
0.4
0.2
0.0
**
Vehicle
ORFV
d
Vehicle
ORFV
4T1
Tumor Volume (mm3)
Vehicle
ORFV
***
Time (days post tumor challenge)
Tumor weight(g)
*
Vehicle ORFV

Supplementary Figure 4. ORFV inhibits tumor growth and triggers pyroptosis in GSDME-low tumor cells a Images from fluorescent microscope displays the changes of EMT6 and CT26 cell morphology and PI uptake after ORFV (MOI=1) or Etoposide (10 μM) treatment for indicated time points. (scale bar: 20 μm). **b** Images from fluorescent microscope displays the changes of 4T1 and B16 cell morphology and PI uptake after ORFV (MOI=1) or Etoposide (10 μM) treatment for indicated time points. (scale bar: 20 μm). **c** 6 weeks old male C57BL/6 mice were s.c. engrafted with B16 cells (1×10^6 cells/mouse). B16 tumors were collected after ORFV i.t. treatment, photographs and weights were shown (n=7) (*P*=0.0098). **d** 6 weeks old female BALB/c mice were s.c. engrafted with 4T1 cells (1×10^6 cells/mouse). 4T1 tumors were collected after ORFV i.t. treatment, photographs, tumor growth curve and weights were shown (n=7) (*P*=0.0002, *P*=0.0415). The above experiments were successfully repeated 2-3 times. *ns*, not significant. * $P<0.05$, ** $P<0.01$, *** $P<0.001$. Two-tailed unpaired Student's t-tests were performed for the statistical analyses in (c and d) and the results were presented as means ± SD.

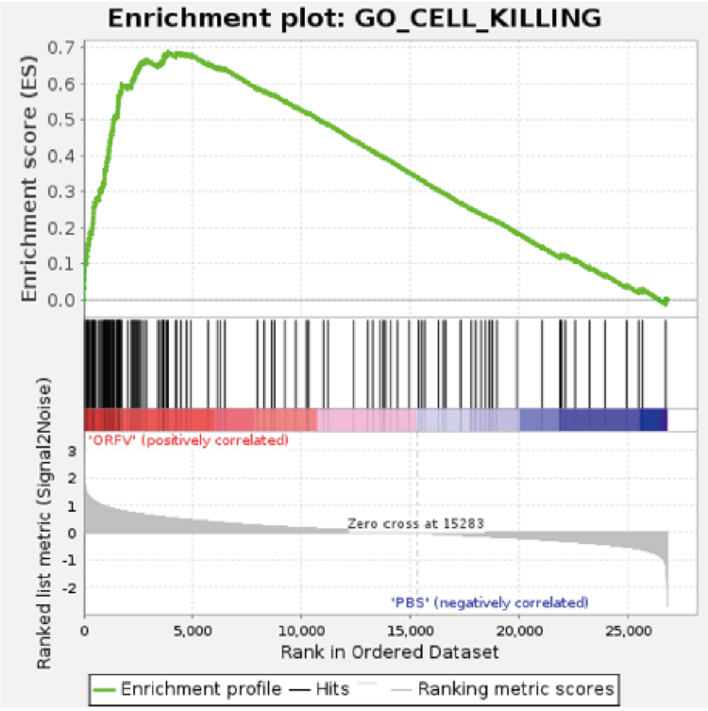

Supplementary Figure 5. ORFV triggers cell killing signaling *in vivo*. GSEA analysis shows the regulation of cell killing related genes in tumor tissues upon ORFV treatment.

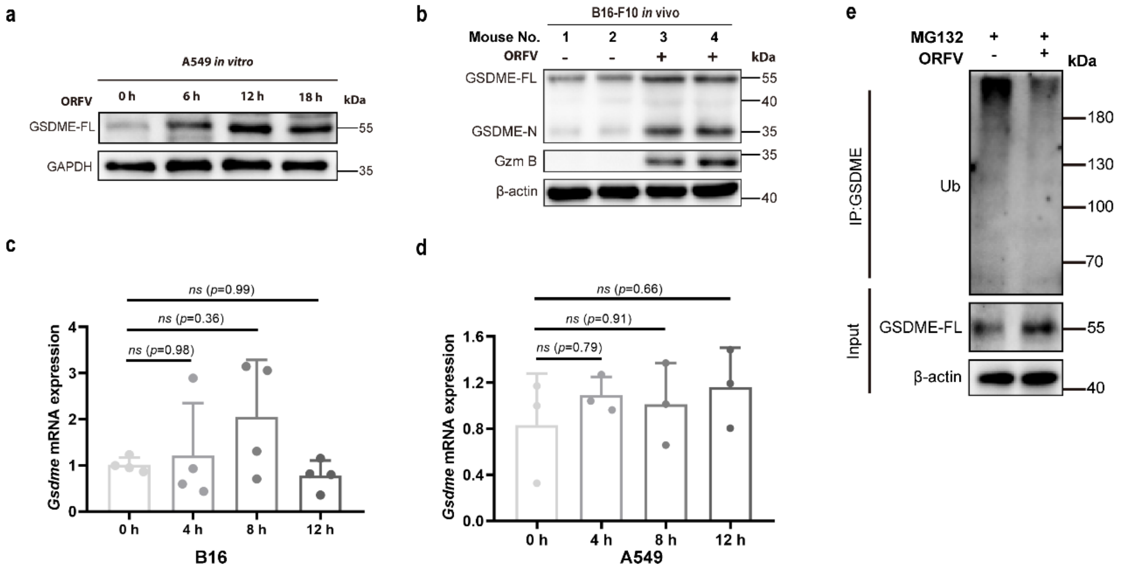

Supplementary Figure 6. ORFV treatment increases GSDME protein level but not mRNA level. a Immunoblots for the detection of GSDME-FL in ORFV-treated A549 cell lysates. **b** Immunoblots for the detection the protein level of GSDME-FL, GSDME-N and GZMB in B16-F10 tumor tissues lysates. **c** RT-qPCR analysis from ORFV treated B16 cells. Relative mRNA level changes of *Gsdme* was displayed. ns, not significant. **d** RT-qPCR analysis from ORFV treated A549 cells. Relative mRNA level changes of *Gsdme* was displayed. **e** Immunoprecipitation analysis of ubiquitination of endogenous GSDME in A549 cells upon ORFV (MOI = 1) in the presence of 10 μM MG132. The above experiments were successfully repeated 2-3 times. *ns*, not significant. Two-tailed unpaired Student's t-tests were performed for the statistical analyses in (c and d) and the results were presented as means ± SD.

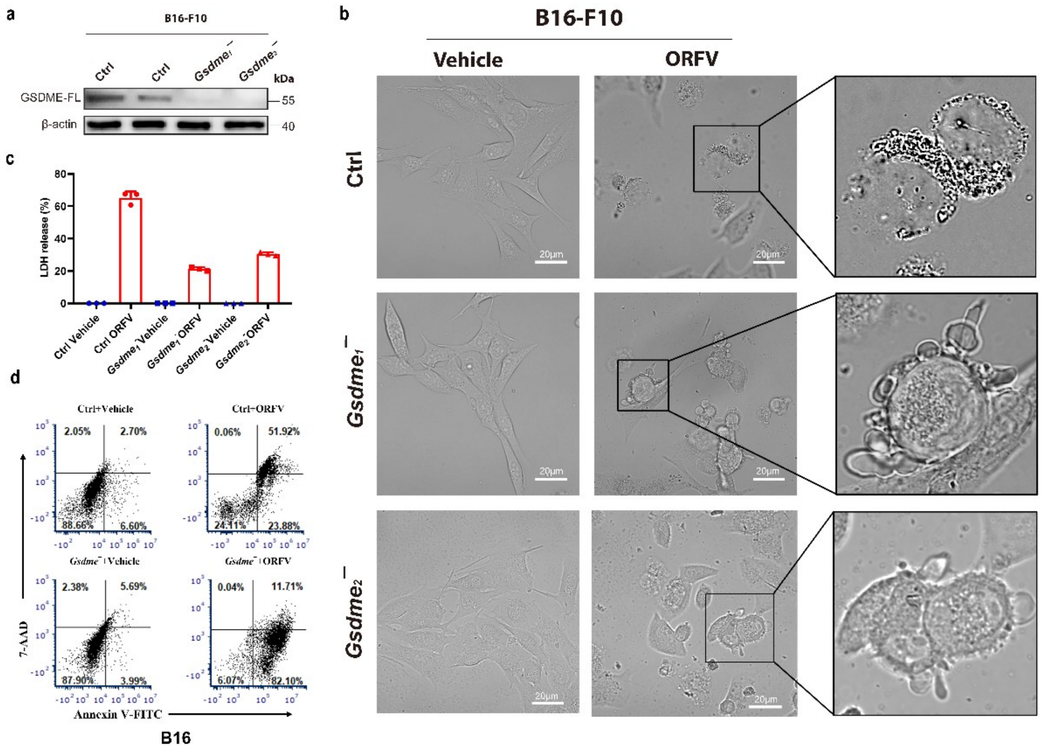

Supplementary Figure 7. GSDME depletion blocks ORFV-induced B16-F10 cells pyroptosis. a Immunoblots for the detection the protein level of GSDME-FL in B16-F10 cell lysates. **b** Images acquired by microscopy display changes of B16-F10 cell morphology upon ORFV challenge (MOI = 1) with or without GSDME depletion (scale bar: 20 μm). **c** LDH release assays were performed with Ctrl or *Gsdme*⁻ B16-F10 cells after ORFV (MOI = 1) treatment for the indicated times (n=3). The above experiments were repeated 2-3 times with similar results and results were presented as means ± SD. **d** FACS of 7-AAD and Annexin V- FITC with or without GSDME depletion B16 cells.

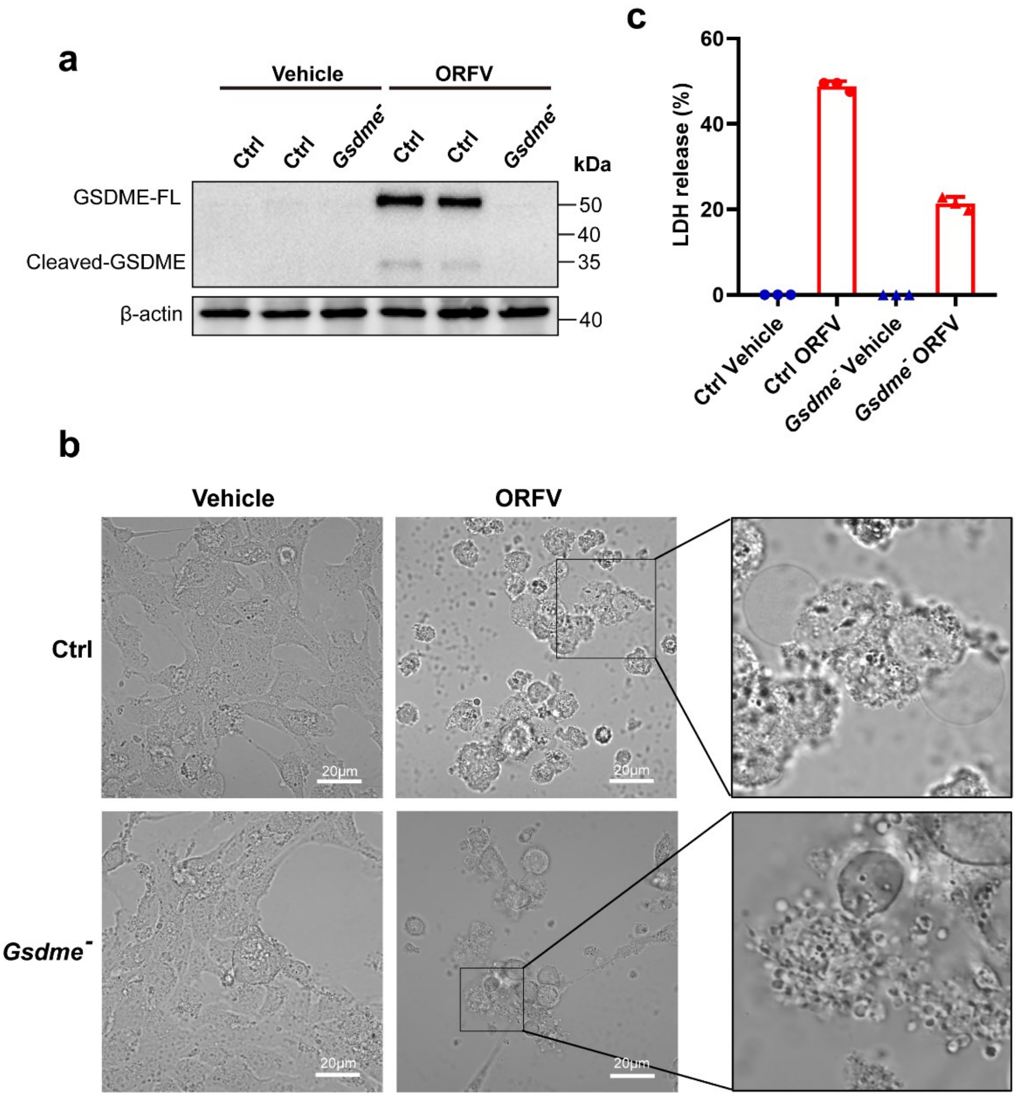

Supplementary Figure 8. GSDME depletion blocks ORFV-induced 4T1 cells pyroptosis. a Immunoblots for the detection the protein level of GSDME-FL in 4T1 cell lysates. **b** Images acquired by microscopy display changes of 4T1 cell morphology upon ORFV challenge (MOI = 1) with or without GSDME depletion (scale bar: 20 μm). **c** LDH release assays were performed with Ctrl or *Gsdme*- 4T1 cells after ORFV (MOI = 1) treatment for the indicated times (n=3). The above experiments were successful repeated 2-3 times. and results were presented as means ± SD in (c).

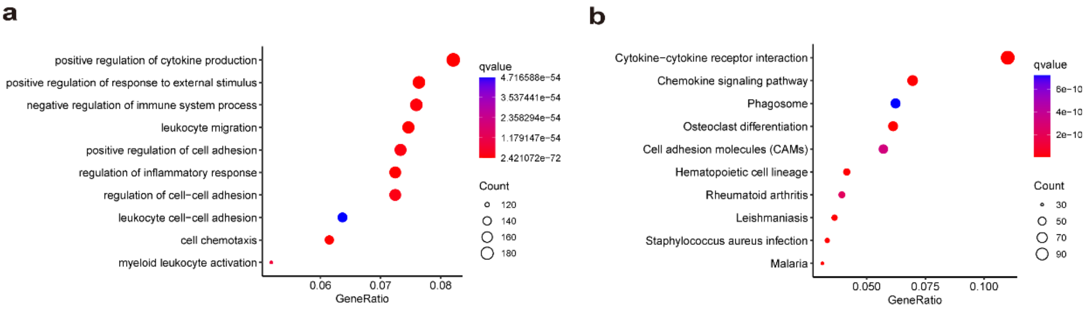

Supplementary Figure 9. ORFV activates inflammatory signaling *in vivo*. a ORFV treatment regulates immune responses and leukocyte migration. GO biological process (BP) analysis displays the top regulated BPs. **b** ORFV treatment regulates cytokine function and chemokine signaling. KEGG analysis displays the top regulated pathways.

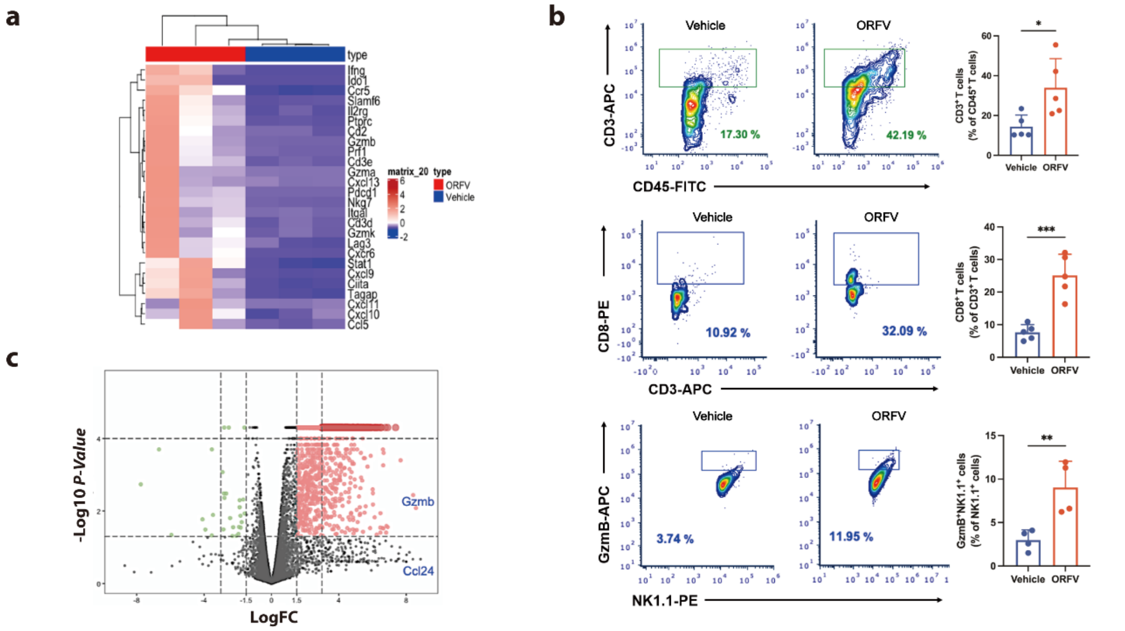

Supplementary Figure 10. ORFV activates antitumor immune response *in vivo*. a Representative heatmap of ORFV-regulated genes based on NanoString PanCancer immune profiling analysis (T cell-inflamed GEPs from 18-gene profile (26)). Three rounds of treatment were performed before tumor collection. **b** FACS analysis for the detection of $CD3^+CD45^+$T cells, $CD8^+CD3^+$T cells and $GzmB^+$ NK cells infiltrated in the tumors. Quantitative analysis results are shown on the right (n=4-5 tumor-bearing mice) (*P*=0.0246, *P*=0.0005, *P*=0.0098)**.** FACS analysis from biological replicate. **c** Volcano plot analyzed from RNA-seq data displays statistical significance (P value) versus magnitude of change (fold change) between vehicle and ORFV-treated tumor tissues, *GzmB* is among the top upregulated transcripts in tumor tissue upon ORFV treatment. Statistical significance was evaluated with Cuffdiff by using two-tailed t-test without adjustment. The above experiments were successful repeated 2-3 times. * *P*<0.05, ** *P*<0.01, ****P*<0.001. Two-tailed unpaired Student's t-test were performed for the statistical analysis in (b, c) and the results were presented as means ± SD.

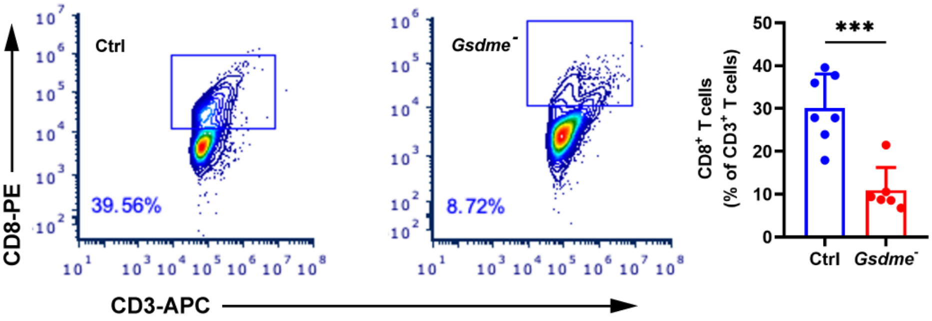

Supplementary Figure 11. GSDME depletion impairs TILs in tumors in the context of ORFV challenge. Mice were engrafted with Ctrl or *Gsdme*$^{-}$ B16 tumor cells, and the mice were i.t. treated with ORFV (1×10^{5} $TCID_{50}$/mouse). FACS analyses for the detection of $CD8^{+}CD3^{+}$ T cells. Quantitative analysis results are shown on the right (n=6-7) (P=0.0004). The above experiments were successful repeated 2-3 times. *** P<0.001, two-tailed unpaired Student's t-test were performed for the statistical analysis, and the results were presented as means ± SD.

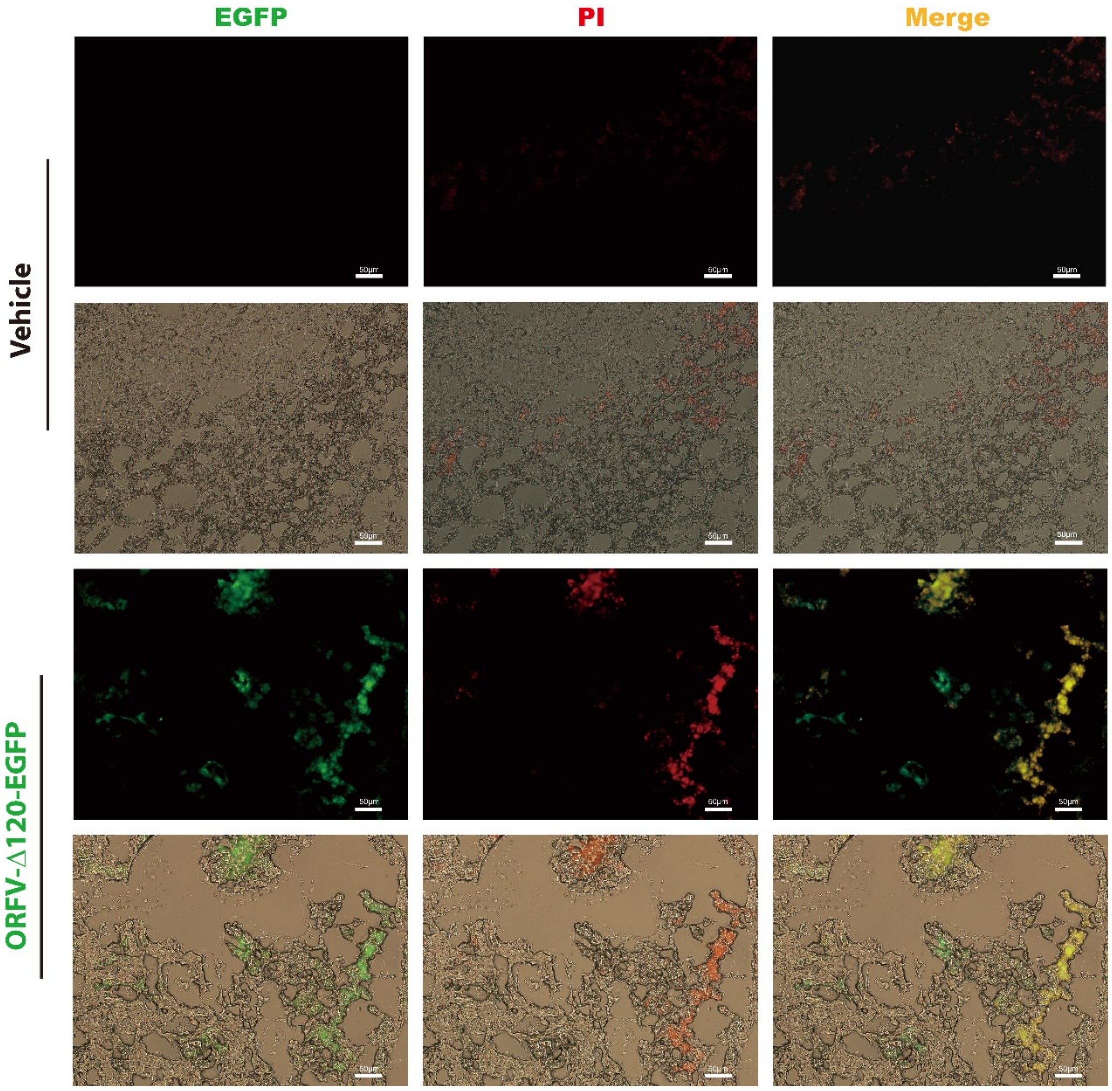

Supplementary Figure 12. ORFV replicates and causes lytic cell death *in vivo*. Images acquired with a fluorescence microscope display ORFV-Δ120-EGFP replication and PI uptake in situ 4T1 tumors (scale bar: 50 μm). The above experiments were successful repeated 2-3 times.

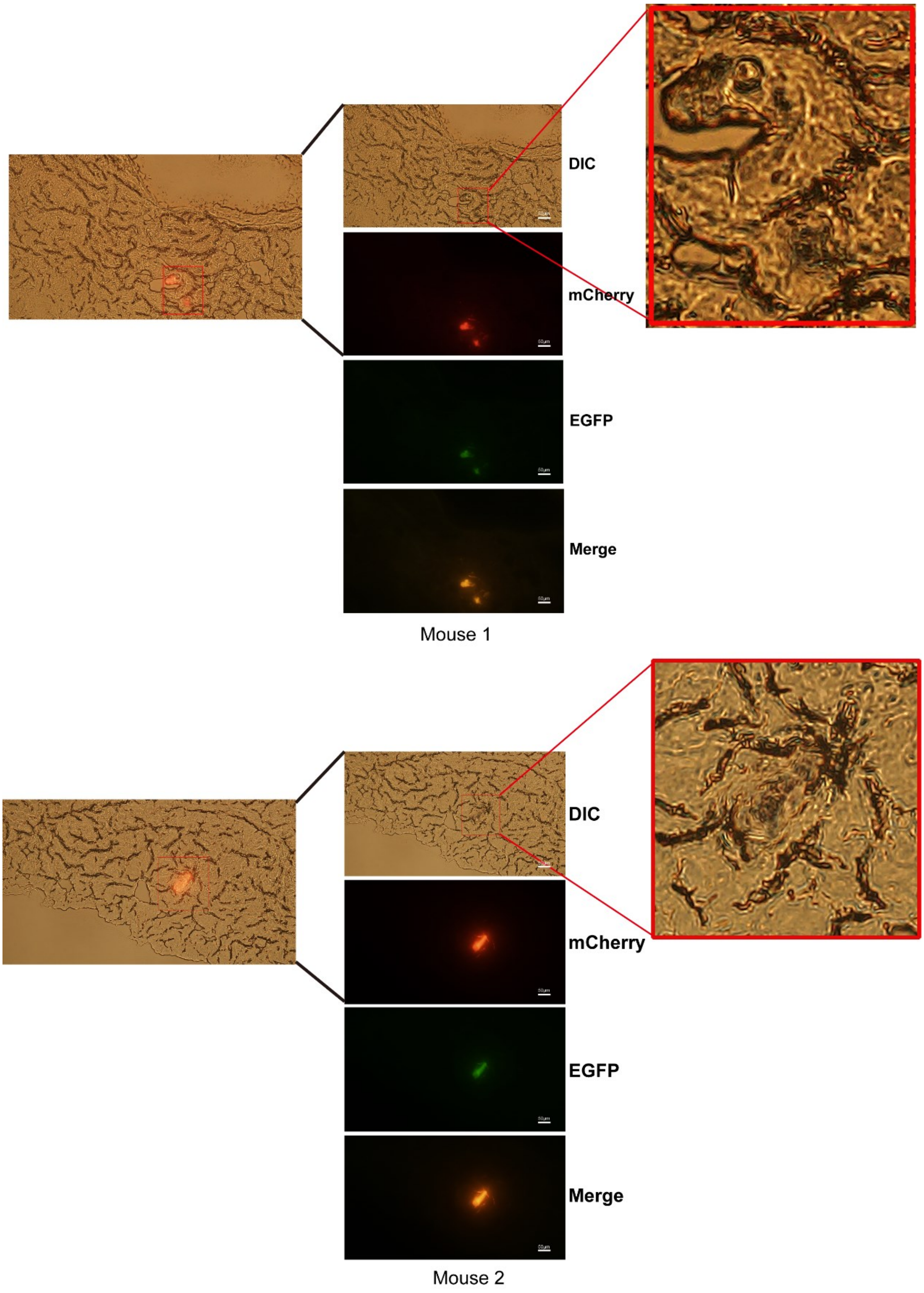

Supplementary Figure 13. ORFV recombinants target metastatic tumor. The B16-F10-mCherry cells have been engrafted i.v. into the mice to establish a metastatic mouse model. After the establishment of the tumors *in vivo*, the mice were treated i.v. with ORFV-Δ120-EGFP. After collecting samples from the lungs, red fluorescence signal (B16-F10-mCherry cells), green fluorescence signal (ORFV-Δ120-EGFP) and black color were found in the same region (scale bar: 50 μm). The above experiments were successful repeated 2-3 times.

Supplementary Table 1 Sequences of the RT-qPCR primers used in this study

GAPDH-F	GGTGAAGGTCGGTGTGAACG
GAPDH-R	CTCGCTCCTGGAAGATGGTG
GSDMD F'	CCATCGGCCTTTGAGAAAGTG
GSDMD R'	ACACATGAATAACGGGGTTTCC
IL18 F'	GACTCTTGCGTCAACTTCAAGG
IL18R'	CAGGCTGTCTTTTGTCAACGA
IL6 F'	TAGTCCTTCCTACCCCAATTTCC
IL6 R'	TTGGTCCTTAGCCACTCCTTC
IL1β F'	GCAACTGTTCCTGAACTCAACT
IL1β R'	ATCTTTTGGGGTCCGTCAACT
casp 1 F'	ACAAGGCACGGGACCTATG
casp 1 R'	TCCCAGTCAGTCCTGGAAATG
casp 8 F'	TGCTTGGACTACATCCCACAC
casp 8 R'	TGCAGTCTAGGAAGTTGACCA
NLRC4 F'	ATCGTCATCACCGTGTGGAG
NLRC4 R'	GCCAGACTCGCCTTCAATCA
GSDME F’	TGCAACTTCTAAGTCTGGTGACC
GSDME R’	CTCCACAACCACTGGACTGAG

Supplementary Methods

B16-F10-mCherry cell line construction. Lentivirus LV-CMV-mCherry-T2A-Puro purchased from Cyagen (Suzhou, China). MOI=1 lentivirus infected B16-F10 cells, after 48 h, preliminary screening of infected cells with 2.5 *μg/mL* puromycin for 3 days. The preliminarily screened cells were further purified by flow cytometry (BD, Aria II). The selected cells were used in animal experiments.
