# Supplementary material for: Oncolytic Parapoxvirus induces Gasdermin E-mediated pyroptosis and activates antitumor immunity
Source: Nat Commun. 2023 Jan 14;14:224. doi: 10.1038/s41467-023-35917-2 (PMC9840172; doi:10.1038/s41467-023-35917-2)
Supplement: Supplementary file 2 — Description of Additional Supplementary Files [file 41467_2023_35917_MOESM2_ESM.pdf]

Description of Additional Supplementary Files

File Name: Supplementary Movie 1
Description: **ORFV induces cell swelling and PI dye uptake in NCI-H226 cells**
NCI-H226 cells were challenged with ORFV (MOI=1) for 16 h, PI dye in the culture medium entered cells as the cell swelled, indicating the destruction of cell membrane.

File Name: Supplementary Movie 2
Description: **EGFP proteins leaked from ORFV-Δ120-EGFP-challenged B16 cells**
B16 cells were challenged with ORFV-Δ120-EGFP (MOI=1) for 26 h, EGFP proteins in the cells leaked into the cell culture medium and the green fluorescence strength from the cells became weaker. Indicating the pore-forming in the membrane of pyroptotic cells.
