# Supplementary material for: Oncolytic Parapoxvirus induces Gasdermin E-mediated pyroptosis and activates antitumor immunity
Source: Nat Commun. 2023 Jan 14;14:224. doi: 10.1038/s41467-023-35917-2 (PMC9840172; doi:10.1038/s41467-023-35917-2)
Supplement: Supplementary file 5 — Reporting Summary [file 41467_2023_35917_MOESM5_ESM.pdf]

nature portfolio

Corresponding author(s): Wenqi He & Jiyu Guan

Last updated by author(s): 12/12/2022

Reporting Summary

Nature Portfolio wishes to improve the reproducibility of the work that we publish. This form provides structure for consistency and transparency in reporting. For further information on Nature Portfolio policies, see our Editorial Policies and the Editorial Policy Checklist.

Statistics

For all statistical analyses, confirm that the following items are present in the figure legend, table legend, main text, or Methods section.

n/a	Confirmed	
☐	☒	The exact sample size (*n*) for each experimental group/condition, given as a discrete number and unit of measurement
☐	☒	A statement on whether measurements were taken from distinct samples or whether the same sample was measured repeatedly
☐	☒	The statistical test(s) used AND whether they are one- or two-sided<br>*Only common tests should be described solely by name; describe more complex techniques in the Methods section.*
☐	☒	A description of all covariates tested
☐	☒	A description of any assumptions or corrections, such as tests of normality and adjustment for multiple comparisons
☐	☒	A full description of the statistical parameters including central tendency (e.g. means) or other basic estimates (e.g. regression coefficient) AND variation (e.g. standard deviation) or associated estimates of uncertainty (e.g. confidence intervals)
☐	☒	For null hypothesis testing, the test statistic (e.g. *F*, *t*, *r*) with confidence intervals, effect sizes, degrees of freedom and *P* value noted<br>*Give P values as exact values whenever suitable.*
☒	☐	For Bayesian analysis, information on the choice of priors and Markov chain Monte Carlo settings
☒	☐	For hierarchical and complex designs, identification of the appropriate level for tests and full reporting of outcomes
☒	☐	Estimates of effect sizes (e.g. Cohen's *d*, Pearson's *r*), indicating how they were calculated

Our web collection on statistics for biologists contains articles on many of the points above.

Software and code

Policy information about availability of computer code

Data collection	OLYMPUS (FV3000) for images and movies collection;<br>OLYMPUS (BX53) for frozen section images collection;<br>Biorad (CFX96) for performing real-time PCR assay;<br>Tanon-5200 for the detection Western Blotting bands;<br>Hitachi (H-9500) for the observation of virus particle morphology;<br>Immunohistochemical analysis are obtained through the PANNORAMIC MIDI II automatic digital slide scanner (3DHISTECH, Budapest, Hungary).<br>Beckman Coulter CytoFLEX for flow cytometry raw data collection.
Data analysis	GraphPad Prism 9 was used to perform statistical analysis and to draw line, column and survival plots;<br>FCS Express 7 was performed to analyze flow cytometry data;<br>Adobe Illustrator CS5 was used to organize figures;<br>Adobe PhotoShop CS5 was used to display photo items;<br>ImageJ (NIH, 1.50i) was used to quantify the intensity of bands from Western Blotting;<br>R (version 4.0.3) software was performed to analyze RNA-seq data.

For manuscripts utilizing custom algorithms or software that are central to the research but not yet described in published literature, software must be made available to editors and reviewers. We strongly encourage code deposition in a community repository (e.g. GitHub). See the Nature Portfolio guidelines for submitting code & software for further information.

Data

Policy information about availability of data

All manuscripts must include a data availability statement. This statement should provide the following information, where applicable:

- Accession codes, unique identifiers, or web links for publicly available datasets
- A description of any restrictions on data availability
- For clinical datasets or third party data, please ensure that the statement adheres to our policy

All data generated or analyzed during this study are included in this manuscript. The RNA seq data (GSE206634) was deposited in Gene Expression Omnibus (GEO) DataSets: https://www.ncbi.nlm.nih.gov/geo/query/acc.cgi?acc=GSE206634. For other data, from this study are available from the corresponding author upon reasonable request.

Human research participants

Policy information about studies involving human research participants and Sex and Gender in Research.

Reporting on sex and gender	Four patient tumor tissues were randomly collected, in which two samples were from male patients and two samples were from female patients.
Population characteristics	There is no population characteristics analysis in the present manuscript.
Recruitment	Four patient tumor tissues were randomly collected. There is no potential self-selection bias or other biases that could affect the results.
Ethics oversight	Ethics Committee of The First Hospital of Jilin University

Note that full information on the approval of the study protocol must also be provided in the manuscript.

Field-specific reporting

Please select the one below that is the best fit for your research. If you are not sure, read the appropriate sections before making your selection.

☒ Life sciences ☐ Behavioural & social sciences ☐ Ecological, evolutionary & environmental sciences

For a reference copy of the document with all sections, see nature.com/documents/nr-reporting-summary-flat.pdf

Life sciences study design

All studies must disclose on these points even when the disclosure is negative.

Sample size	In the present study, we did not perform sample-size calculation. The choose of sample sizes was based on the current literature standards. The sample sizes were sufficient for statistical analysis.
Data exclusions	No data exclusions.
Replication	The results of the in vivo studies were successfully repeated for two or three times; the results of cell or tumor tissue-based studies were successfully repeated for three or more times.
Randomization	6-8 weeks old mice with similar weight and healthy status were randomly grouped. Gender selection was based on the type of tumor cell lines. Randomization was not used for in vitro and ex vivo experiments.
Blinding	Investigators were not blinded to perform in vitro, ex vivo, animal in vivo experiments as well as biochemical assays.

Reporting for specific materials, systems and methods

We require information from authors about some types of materials, experimental systems and methods used in many studies. Here, indicate whether each material, system or method listed is relevant to your study. If you are not sure if a list item applies to your research, read the appropriate section before selecting a response.

Materials & experimental systems

n/a	Involved in the study	
☐	☒	Antibodies
☐	☒	Eukaryotic cell lines
☒	☐	Palaeontology and archaeology
☐	☒	Animals and other organisms
☒	☐	Clinical data
☒	☐	Dual use research of concern

Methods

n/a	Involved in the study	
☒	☐	ChIP-seq
☐	☒	Flow cytometry
☒	☐	MRI-based neuroimaging

Antibodies

Antibodies used

Anti-GSDME (Abcam, Rabbit monoclonal [EPR19859], cat#ab215191, 1:1000); anti-HMGB1 (Abcam, Rabbit polyclonal to HMGB1, cat#ab18256, 1 μg/ml); anti-GZMB (Abcam, Rabbit monoclonal [EPR22645-206], cat#ab255598, 1:1000); Alexa Fluor®647-conjugated anti-melanoma gp100 (Abcam, Rabbit monoclonal [EP4863(2)], cat#ab246730, 1:5000); Anti-Caspase 3 (Cell Signaling Technology, Rabbit polyclonal to Caspase 3, cat#9662, 1:1000); anti-Ubiquitin (P4D1) Mouse mAb (Cell Signaling Technology, mouse monoclonal P4D1, cat#3936T, 1:1000); anti-PARP (Cell Signaling Technology, Rabbit monoclonal 46D11, cat#9532, 1:1000); anti-GAPDH (Cell Signaling Technology, Rabbit monoclonal D16H11, cat#5174, 1:1000); anti-β-actin (Cell Signaling Technology, Rabbit monoclonal D6A8, cat#8457, 1:1000); anti-PD-1 (Cell Signaling Technology, Rabbit monoclonal D7D5W, cat#84651, 1:1000); anti-mouse IgG HRP-linked antibody (Cell Signaling Technology, cat#7076, 1:3000); anti-rabbit IgG HRP-linked antibody (Cell Signaling Technology, cat#7074, 1:3000); Anti-mouse CD16/32 (BioLegend, Rabbit Monoclonal S17011E, cat#156603, 0.25 μg per 106 cells in a volume of 100 μl); FITC-conjugated anti-mouse CD45 (BioLegend, Rabbit Monoclonal I3/2.3, cat#147710, ≤0.25 μg per million cells in 100 μl volume); APC-conjugated anti-mouse CD3 (BioLegend, Rabbit Monoclonal 17A2, cat#100236, ≤0.5 μg per million cells in 100 μl volume); PE-conjugated anti-mouse CD8a (BioLegend, Rabbit Monoclonal 53-6.7, cat#100708, ≤ 0.25 μg per 106 cells in 100 μl volume); APC-conjugated anti-mouse CD8a (BioLegend, Rabbit Monoclonal 53-6.7,cat#100712, ≤ 0.25 μg per 106 cells in 100 μl volume); APC anti-human/mouse Granzyme B (BioLegend, Mouse Monoclonal QA16A02, cat#372204, 5 μl per million cells in 100 μl staining volume); PE anti-human/mouse Granzyme B Recombinant (BioLegend, Mouse Monoclonal QA16A02, cat#372207, 5 μl per million cells in 100 μl staining volume); InVivoMab anti-mouse PD-1 (CD279) (BioXCell, Rabbit Monoclonal 29F.1A12, cat#BE0273, 5 mg/kg); InVivoMab anti-mouse CD8α (BioXCell, Rabbit Monoclonal 2.43, cat#BE0061, 5 mg/kg)

Validation

All antibodies were verified by manufacturers and widely used in the published literatures. A full reference list can be found on the official website of the manufacturer. The above antibodies are used in western blotting detection, flow cytometry and mouse in vivo experiments.
Anti-GSDME (Knockout validated shown on the website, data shown in the manuscript PubMed: 30976076); anti-HMGB1(Knockout validated shown on the website, data shown in the manuscript PubMed: 33420008); anti-GZMB(recombinant antibody, the specificity has been confirmed by the manufacturer, data shown in the manuscript PubMed: 34454491); Alexa Fluor®647-conjugated anti-melanoma gp100(recombinant antibody, the specificity has been confirmed by the manufacturer); Anti-Caspase 3(Knockout validated shown on the website, data shown in the manuscript PubMed: 35505004); anti-Ubiquitin (P4D1) Mouse mAb(data shown in the manuscript PubMed: 36288705); anti-PARP(Knockout validated shown on the website, data shown in the manuscript PubMed: 36257929); anti-GAPDH(data shown in the manuscript PubMed: 36302906); anti-β-actin(data shown in the manuscript PubMed: 36103834); anti-PD-1(recombinant antibody, the specificity has been confirmed by the manufacturer, data shown in the manuscript PubMed:35304461);Anti-mouse CD16/32(FC-Quality tested shown on the website, data shown in the manuscript PubMed: 32905782); FITC-conjugated anti-mouse CD45(FC-Quality tested shown on the website, data shown in the manuscript PubMed:29861387); APC-conjugated anti-mouse CD3(FC-Quality tested shown on the website, data shown in the manuscript PubMed:29160310); PE-conjugated anti-mouse CD8a(FC-Quality tested shown on the website, data shown in the manuscript PubMed:29160310); APC-conjugated anti-mouse CD8a(FC-Quality tested shown on the website, data shown in the manuscript PubMed:29255233); APC anti-human/mouse Granzyme B(FC-Quality tested shown on the website, data shown in the manuscript PubMed:30388456); PE anti-human/mouse Granzyme B Recombinant(FC-Quality tested shown on the website, data shown in the manuscript PubMed:30595553); InVivoMab anti-mouse PD-1 (CD279)(functional tested shown on the website); InVivoMab anti-mouse CD8α(functional tested shown on the website).

Eukaryotic cell lines

Policy information about cell lines and Sex and Gender in Research

Cell line source(s)

Tumor cell lines were obtained from National Collection of Authenticated Cell Cultures (ACHN, NCI-H226, A549, 4T1, B16, B16-F10, CT26, RAW264.7) and Procell (Wuhan, China) (EMT6). Primary ovine fetal turbinate (OFTu) cells were isolated and cultured in our laboratory.

Authentication

All cell lines have been identified for authenticity by the supplier, details can be found on their official website.
ACHN, NCI-H226 and A549 were authenticated by morphology and STR profiling; 4T1, B16, B16-F10, CT26, RAW264.7 and EMT6 was authenticated by STR profiling; OFTu was authenticated by morphology.

Mycoplasma contamination

The cell lines were determined to be free of mycoplasma contamination

Commonly misidentified lines
(See ICLAC register)

No commonly misidentified lines were used.

Animals and other research organisms

Policy information about studies involving animals; ARRIVE guidelines recommended for reporting animal research, and Sex and Gender in Research

Laboratory animals	All animals used in this study were chosen randomly. All animals were kept in a standard laboratory and fed sterile food (Chang Sheng Biotechnology, China) and water. The laboratory was a pathogen-free conditions with central air that was controlled by a thermostat (25℃), relative humidity between 40-60 rH, and provide 12 hours light daily.
Wild animals	No wild animals were used.
Reporting on sex	Female BALB/c mice were used to graft 4T1 or EMT-6 cells;<br>Male C57BL/6 mice were used to graft B16 or B16-F10 cells.
Field-collected samples	No field-collected samples were used.
Ethics oversight	The experimental protocol was approved by the ethics committee in the College of Veterinary Medicine of Jilin University.

Note that full information on the approval of the study protocol must also be provided in the manuscript.

Flow Cytometry

Plots

Confirm that:

☒ The axis labels state the marker and fluorochrome used (e.g. CD4-FITC).

☒ The axis scales are clearly visible. Include numbers along axes only for bottom left plot of group (a 'group' is an analysis of identical markers).

☒ All plots are contour plots with outliers or pseudocolor plots.

☒ A numerical value for number of cells or percentage (with statistics) is provided.

Methodology

Sample preparation	Tumor tissues from mice were weighed, minced into small pieces and ground to make cell suspensions in PBS. A 70-µm cell strainer was used to filter the cells to generate a single-cell suspension. The cells were washed with FACS buffer by centrifugation and resuspended in FACS buffer for further staining. Details for sample preparation can be found in the Method section in the manuscript.
Instrument	Beckman Coulter CytoFLEX & BD FACS Aria II
Software	FCS Express 7 software
Cell population abundance	For all studies, 10,000-50,000 cells were collected per sample.
Gating strategy	The gating strategy was according to the distribution of fluorescent antibody-labeled cell population. Individual figures have shown the detailed gating strategy.

☐ Tick this box to confirm that a figure exemplifying the gating strategy is provided in the Supplementary Information.
